# Global Burden of Chronic Obstructive Pulmonary Disease and Heart Failure Comorbidity in Older Adults: An Analysis of the Global Burden of Disease Study

**DOI:** 10.14336/AD.2025.1044

**Published:** 2026-09-28

**Authors:** Jian Kang, Huiqing Xu, Xiaojing Deng, Yeping Bian, Tianrui Deng, Yunting Xu, Guofeng Ao, Jian Xu, Fei Xu

**Affiliations:** ^1^Department of Intensive Care Medicine, The First Affiliated Hospital with Nanjing Medical University, Nanjing, China.; ^2^Department of Primary Healthcare, Nanjing Municipal Center for Disease Control and Prevention Affiliated to Nanjing Medical University, Nanjing, China.; ^3^Department of Epidemiology, Nanjing Medical University School of Public Health, Nanjing, China.; ^4^Department of Intensive Care Medicine, Geriatric Hospital of Nanjing Medical University, Nanjing, China.

**Keywords:** Chronic obstructive pulmonary disease, heart failure, prevalence, years lived with disability, Global Burden of Diseases, comorbidity

## Abstract

Heart failure (HF) is a common comorbidity for older patients of chronic obstructive pulmonary disease (COPD). This study aimed to investigate the global burden of COPD-HF comorbidity among older adults from 1990 to 2021 and make a prediction till 2050. Data on prevalence and years lived with disability (YLDs) for COPD-HF comorbidity among older adults aged 60 or above were obtained from the Global Burden of Diseases Study 2021. Absolute number and age-standardized rate (ASR) per 100,000 individuals were used to compare the disease burden by sex, age, severity and region. Temporal trends in ASR from 1990 to 2021 were analyzed using Joinpoint models, while Bayesian age-period-cohort models were introduced to project ASR till 2050. From 1990 to 2021, global prevalent cases and YLDs of COPD-HF comorbidity among older adults increased, and ASRs also exhibited a sustained increase. The disease burden will continue to rise till 2050. Moreover, the comorbidity burden was higher in males than females, and increased with age. Additionally, severe and treated cases were the predominant subtypes of this comorbidity. Low and middle socio-demographic index (SDI) regions tended to bear higher disease burden. The global burden of COPD-HF comorbidity was substantially heavy with a consistent rising trend from 1990 to 2021 in older adults. The disease burden will rise till 2050. Disparities of the disease burden existed in sex, SDI, and geographic region worldwide. This study has implications for re-allocating resources to early identification and effective treatment of COPD, HF, and COPD-HF comorbidity.

## INTRODUCTION

Chronic obstructive pulmonary disease (COPD) is a common chronic respiratory disorder, characterized by persistent respiratory symptoms and progressive airflow limitation caused by airway or/and alveolar abnormalities [1]. Typically, acute COPD exacerbations are accompanied by marked dynamic hyperinflation [2]. This necessitates a rise in cardiac output to fulfill increased oxygen requirements, potentially exacerbating pulmonary hypertension and augmenting right ventricular afterload, which may progress to right heart failure (HF) [3]. Therefore, for COPD individuals, particularly those older patients, most of them will develop mild to moderate pulmonary hypertension and experience HF if they are not timely and adequately treated [4, 5].

Both COPD and HF are leading causes of global morbidity and mortality [6, 7]. Moreover, they frequently coexist, and impose substantial disease burden on COPD individuals [8-10]. Among COPD patients, the prevalence of HF varies between 7.8% and 27.6% [11-13], whereas the prevalence of COPD ranges from 9% to 52% in HF patients [14]. Additionally, the prevalence of COPD-HF comorbidity increases substantially with age [15, 16]. As the aging population rapidly grows, early warnings of COPD-HF comorbidity deserve particular attention in older adults aged 60 years or above. However, most existing studies focus separately on the single disease and fail to address the issue of COPD-HF comorbidity among older adults [17].

Presently, COPD-HF comorbidity has been emerging as a major global health concern for older adults, which warrant that a comprehensive evaluation of its epidemiological burden and trajectory modeling becomes imperative. The Global Burden of Diseases, Injuries, and Risk Factors Study (GBD) initiative, with its systematically compiled global data and hierarchically structured models, offers a robust framework for such an assessment [18]. Using data from the latest GBD 2021 database, this study aimed to analyze the global trends in prevalence and disability of COPD-HF comorbidity among older adults aged 60 years or above from 1990 to 2021, and make a prediction till 2050 worldwide.

## MATERIALS AND METHODS

### Data sources

The data on COPD-HF comorbidity among adults aged 60 years or above were obtained from the publicly available GBD 2021 dataset [18]. The GBD 2021 dataset was derived from over 100,000 data sources, incorporating population registries, health surveys, and clinical data. All the data from different sources were then analyzed using unified disease definitions and advanced statistical modeling techniques. The analytical framework includes: 1) data collection and integration: combining multiple data sources, including health records and surveys; 2) statistical modeling and estimation: applying hierarchical regression models and Bayesian meta-regression (DisMod-MR 2.1) to adjust for uncertainty; and 3) attributable risk analysis: employing comparative risk assessment (CRA) methodology to evaluate the impact of different risk factors and adjust for confounding variables.

This dataset also employed standardized approaches to estimate health metrics such as prevalence, incidence, mortality, and disability-adjusted life years (DALYs) in terms of both annual case counts and age-specific rates per 100,000 population [18]. For each metric, the estimates were derived from 1000 posterior draws generated by the GBD algorithm, with the final values calculated as the mean of draw level estimates along with 95% uncertainty intervals (UIs) represented by the 2.5^th^ and 97.5^th^ percentiles of ordered draws [18]. More details on the measurement method and modeling framework of GBD 2021 were published elsewhere [18] (Data Source: Institute for Health Metrics and Evaluation. Used with permission. All rights reserved.).

### Participants

In this study, older adults were defined as individuals aged 60 years or above. The study participants were those global older adults between 1990 and 2021. Participant’s personal information on age and sex was also obtained from GBD 2021 dataset. Due to publicly available de-identified data analyzed, the ethics approval was exempted for the present study.

### Definitions of HF and COPD

HF case was determined by clinical signs and symptom-based criteria, such as that outlined in the Universal Definition and Classification of Heart Failure [19]. In GBD 2021 dataset, individuals were defined as HF patients if they met stage C or D criteria recommended by American College of Cardiology/American Heart Association (ACC/AHA) [18]. In this study, HF was limited to right heart functional impairment due to COPD-inducing overload [18, 20].

COPD was identified using forced expiratory volume in one second (FEV_1_) and forced vital capacity (FVC) based on post-bronchiectasis spirometry test [21]. An individual with the value of FEV_1_/FVC< 0.7 was determined as a COPD patient [21]. Additionally, in GBD 2021, the cases of COPD were defined clearly with the International Classification of diseases (ICD) versions 9 and 10 codes: the codes 491-492 and 496 from ICD-9, and J41-J44 and J47 from ICD-10 [22].

In GBD dataset, the data sources contained the underlying causes of death and all ICD codes in the causal chain. Therefore, it allowed us to be able to link HF to COPD and determine COPD-HF comorbidity. Firstly, the overall burden of HF was estimated using DisMod-MR 2.1 (a Bayesian meta-regression tool). Then, the burden of COPD-HF comorbidity was calculated by limiting the burden of HF to COPD using proportion equation [18, 20].

The outcome variables referred to burden of COPD-HF comorbidity among global older adults aged 60 years or above, specifically including number of prevalent case, age-standardized prevalence, years lived with disability (YLDs) and age-standardized YLDs rate in this study. The choice of indicators primarily depended on the availability of data in the GBD 2021 dataset, so neither incidence nor mortality of COPD-HF comorbidity was included in the analysis [18]. YLDs, the multiplication of prevalence and disability weight of COPD-HF comorbidity, were used to quantify the burden caused by COPD-HF comorbidity during a specified period [18].

### Explanatory variables

A number of explanatory variables were analyzed in the current study, including age, sex, severity of COPD-HF comorbidity, location, socio-demographic index (SDI), and calendar year. SDI is a composite measure reflecting the area-level status of social and economic development, encapsulating income per capita, mean years of schooling over the age of 15 years, and fertility rate under the age of 25 years. As a continuous measure, this index ranges from 0 to 1, with higher value indicating higher development level. In the analysis, SDI was quintiled into five categories (low, low-middle, middle, high-middle, and high SDI) to present different socio-economic development levels. In analysis, SDI categories were assigned to participants at the country level.

Participants were categorized as men or women. Moreover, they were also classified into eight subgroups with a 5-year interval by age: 60-64, 65-69, 70-74, 75-79, 80-84, 85-89, 90-94, or 95+ years old. Additionally, based on the severity of clinical symptoms, COPD-HF comorbidity was sub-divided into four severity categories: treated, mild, moderate, or severe. All the global 204 countries or territories were categorized into - a) 21 geographical regions per World Health Organization (WHO) classification (Supplementary Table 1), and b) quintiles of SDI - to examine COPD-HF burden by region and socio-economic development status (Supplementary Table 2).

**Figure 1.**
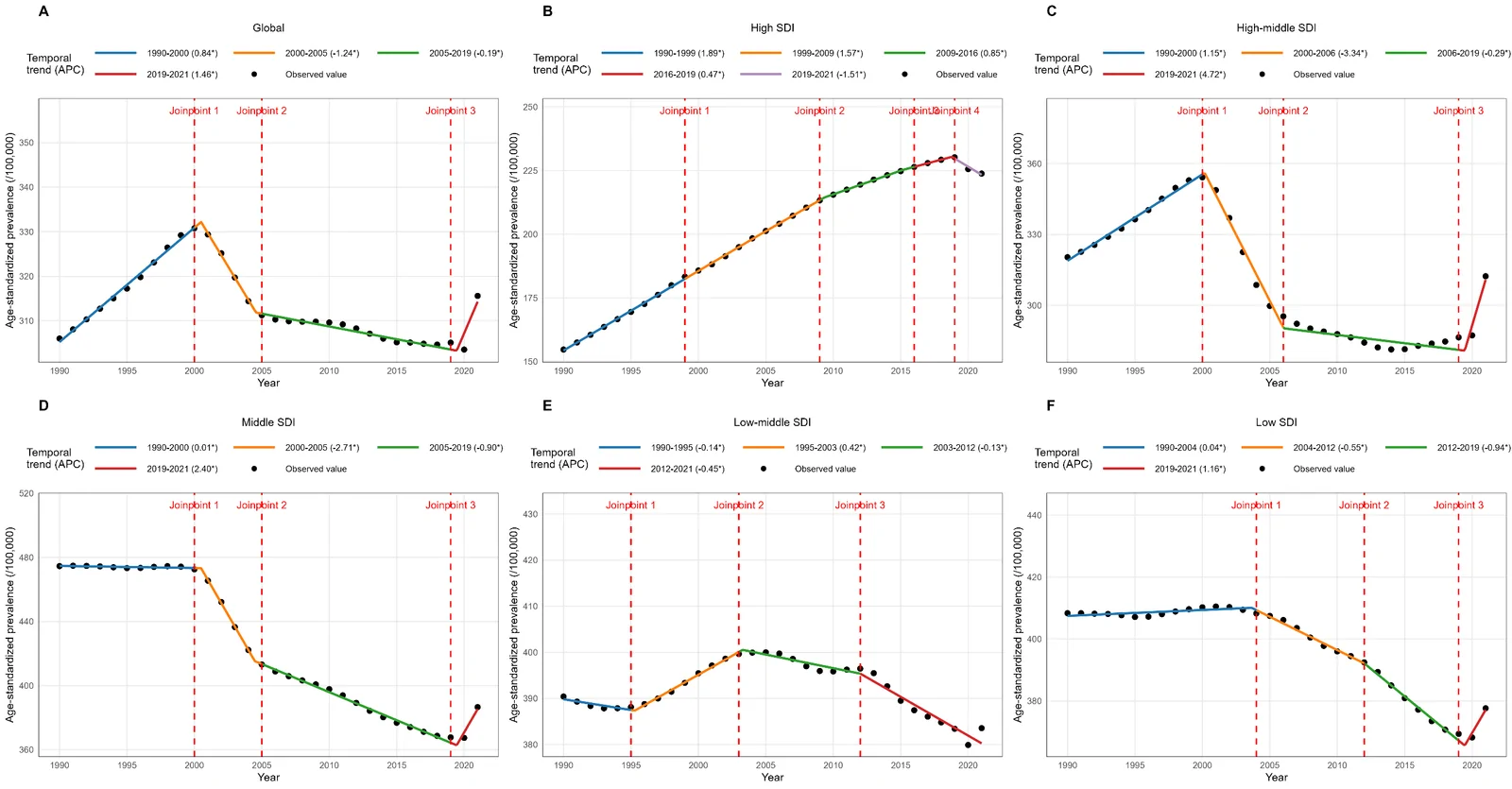
**Joinpoint regression analysis of age-standardized prevalence for COPD-HF comorbidity in adults aged 60 and older from 1990 to 2021 globally and by SDI quintiles**. (**A**) Trends in the globe; (B) Trends in high SDI region; (C) Trends in high-middle SDI region; (D) Trends in middle SDI region; (E) Trends in low-middle SDI region; (F) Trends in low SDI region. *Indicates that the APC is significantly different from zero at the alpha = 0.05 level. COPD, chronic obstructive pulmonary disease; HF, heart failure; SDI, socio-demographic index; APC, annual percentage change

### Statistical analysis

First, the Joinpoint Regression Program (version 5.1.0.0) was employed to analyze trends in disease burden using the logarithmically scaled age-standardized rates (ASRs) as the dependent variable and calendar years as the independent variable [23]. The trends were fit through a log-linear model to identify line segments or joinpoints [23]. The measures for assessing the trends included the annual percentage change (APC), average annual percentage change (AAPC), and their 95% confidence intervals (CIs) [24]. The APC represents the average annual change rate of disease burden during specific periods, while the AAPC, derived as the geometrically weighted average of APCs, reflects the average annual change rate over the entire study period [24]. If the 95%CI of the AAPC is entirely above 0, it suggests a statistically significant increasing trend; if entirely below 0, it demonstrates a statistically significant decreasing trend; and if it includes 0, it suggests no significant change in trend during the period.

Next, the ASRs of prevalence and YLDs for COPD-HF comorbidity were predicted until 2050 using Bayesian age-period-cohort (BAPC) model. The BAPC model has been examined having excellent accuracy in predicting disease burden [25, 26]. Briefly, the model estimates the expected rates based on a logarithmic linear Poisson model with multiplicative effects of age structure, calendar year, and population size [27]. Moreover, the "integrated nested Laplace approximations" package was introduced to avoid mixing and convergence issues, as it could approximate the posterior marginal distributions directly [26].

Finally, Spearman’s correlation coefficients (*r*) were introduced to quantify the overall monotonic relationship between COPD-HF comorbidity burden in older adults and SDI, and a smoothing splines model was used to visualize the associations. The LOESS (locally estimated scatterplot smoothing) algorithm was applied to fit trend curves, based on the ASRs, SDI and time-span parameters across all locations [28]. All statistical analyses were conducted using R version 4.3.3.

## RESULTS

### Global burden of COPD-HF comorbidity in overall older adults from 1990 to 2021 and its projection till 2050

Table 1 presented the overall burden of COPD-HF comorbidity in older adults from 1990 to 2021 worldwide. During this period, there was a two-fold increase in prevalent cases of COPD-HF comorbidity in older adults (from 1,339,243.52 in 1990 to 3,303,441.39 in 2021), while the YLDs rose from 118,724.14 to 293,134.50. For ASR, both the global prevalence (306.01 to 315.58 per 100,000 population; AAPC: 0.08; 95% CI: 0.03, 0.13) and YLDs rates (27.05 to 27.98 per 100,000 population; AAPC: 0.09; 95% CI: 0.04, 0.14) of COPD-HF comorbidity showed a slight increase in older adults from 1990 to 2021. The significant increasing temporal trends were observed in ASR of either prevalence or YLDs of COPD-HF comorbidity over the past 32 years (Fig. 1 and Supplemenatary Fig. 1).

**Table 1:** Number of prevalent cases, YLDs and ASRs of prevalence and YLDs for COPD-HF comorbidity in older adults (aged 60+ years) in 1990 and 2021, and the AAPC from 1990 to 2021 by region

Location	Prevalence					YLDs				
	Number (95% UI), 1990	ASR per 100,000 (95% UI), 1990	Number (95% UI), 2021	ASR per 100,000 (95% UI), 2021	1990-2021 AAPC (95% CI)	Number (95% UI), 1990	ASR per 100,000 (95% UI), 1990	Number (95% UI), 2021	ASR per 100,000 (95% UI), 2021	1990-2021 AAPC (95% CI)
**Global**	1339243.52 (881816.15, 1926534.54)	306.01 (201.96, 440.43)	3303441.39 (2053914.00, 4985427.85)	315.58 (196.30, 475.96)	0.08 (0.03, 0.13)	118724.14 (68246.13, 186477.38)	27.05 (15.63, 42.44)	293134.50 (160506.04, 482034.29)	27.98 (15.34, 45.99)	0.09 (0.04, 0.14)
**SDI regions**										
**High SDI**	224466.20 (141605.50, 331772.45)	154.71 (97.85, 228.76)	669717.58 (432004.49, 989497.29)	223.81 (144.70, 329.87)	1.19 (1.14, 1.24)	20204.78 (10964.64, 32945.74)	13.92 (7.58, 22.71)	60177.03 (33457.27, 98052.57)	20.14 (11.21, 32.77)	1.19 (1.14, 1.24)
**High-middle SDI**	359598.88 (237754.10, 510511.13)	320.35 (212.78, 454.61)	780046.95 (478675.32, 1186759.86)	312.32 (191.74, 475.21)	-0.11 (-0.26, 0.04)	31984.34 (18318.83, 49674.20)	28.41 (16.41, 44.03)	69465.34 (36824.50, 114355.13)	27.79 (14.76, 45.75)	-0.10 (-0.24, 0.04)
**Middle SDI**	465894.15 (307181.93, 661934.97)	474.54 (314.47, 674.91)	1139020.76 (690839.13, 1740388.12)	386.55 (235.30, 588.83)	-0.69 (-0.76, -0.62)	41293.88 (23879.23, 64025.79)	41.83 (24.49, 64.55)	100931.24 (53823.96, 166978.04)	34.16 (18.32, 56.38)	-0.68 (-0.75, -0.61)
**Low-middle SDI**	210240.63 (138506.94, 306692.24)	390.40 (256.75, 569.35)	544416.87 (342000.51, 825245.01)	383.55 (240.47, 580.54)	-0.08 (-0.13, -0.04)	18316.02 (10497.07, 29631.89)	33.78 (19.48, 54.43)	47622.30 (25822.98, 80224.58)	33.39 (18.17, 56.17)	-0.06 (-0.11, -0.01)
**Low SDI**	78416.78 (46038.02, 125470.05)	408.34 (242.36, 647.41)	168852.22 (96675.04, 278120.43)	377.63 (216.56, 619.30)	-0.26 (-0.31, -0.22)	6869.01 (3481.40, 11821.77)	35.42 (18.36, 60.38)	14814.62 (7445.79, 26349.11)	32.91 (16.65, 58.29)	-0.25 (-0.29, -0.21)
**Geographical regions**										
**Andean Latin America**	2677.48 (1873.33, 3711.08)	125.66 (87.77, 174.54)	9330.88 (6270.57, 13431.30)	134.84 (90.65, 194.01)	0.27 (0.22, 0.32)	236.16 (133.26, 369.60)	11.06 (6.27, 17.26)	827.04 (460.50, 1334.66)	11.95 (6.66, 19.26)	0.29 (0.23, 0.34)
**Australasia**	7018.32 (4680.08, 9922.73)	227.41 (151.33, 322.71)	27085.31 (19784.46, 35928.64)	362.09 (264.95, 479.27)	1.53 (1.47, 1.59)	632.72 (333.77, 1020.80)	20.48 (10.81, 33.14)	2430.89 (1437.51, 3619.67)	32.52 (19.24, 48.45)	1.53 (1.46, 1.60)
**Caribbean**	3049.84 (2110.65, 4256.31)	100.86 (69.43, 141.55)	9798.20 (6661.44, 13982.88)	143.68 (98.10, 204.33)	1.16 (1.12, 1.20)	273.12 (154.11, 436.11)	9.00 (5.10, 14.37)	874.54 (487.14, 1396.26)	12.83 (7.17, 20.45)	1.18 (1.10, 1.26)
**Central Asia**	5033.11 (3121.41, 7491.37)	99.63 (61.53, 149.19)	5138.85 (3000.20, 7869.95)	62.33 (35.93, 96.13)	-1.47 (-1.55, -1.39)	448.56 (232.04, 735.56)	8.86 (4.57, 14.59)	460.99 (228.30, 787.48)	5.57 (2.75, 9.54)	-1.46 (-1.54, -1.37)
**Central Europe**	15384.40 (9532.32, 23125.10)	86.01 (53.29, 129.90)	26558.02 (17076.56, 39176.95)	85.39 (54.88, 125.99)	-0.03 (-0.09, 0.02)	1384.65 (724.63, 2327.76)	7.72 (4.05, 13.01)	2390.50 (1297.01, 3970.39)	7.69 (4.17, 12.77)	-0.02 (-0.11, 0.07)
**Central Latin America**	17459.78 (11695.31, 24873.85)	209.97 (140.71, 299.42)	64334.76 (41647.89, 95264.77)	221.43 (143.55, 327.19)	0.16 (0.13, 0.19)	1545.53 (895.93, 2388.19)	18.51 (10.80, 28.53)	5714.26 (3203.72, 9384.44)	19.65 (11.05, 32.24)	0.18 (0.15, 0.21)
**Central Sub-Saharan Africa**	5487.58 (2546.58, 10245.26)	311.84 (144.93, 580.46)	10491.67 (4761.35, 20031.91)	247.49 (112.62, 470.30)	-0.75 (-0.80, -0.71)	489.06 (195.59, 979.65)	27.54 (11.23, 54.64)	933.38 (369.99, 1902.96)	21.91 (8.86, 44.58)	-0.74 (-0.80, -0.69)
**East Asia**	655831.01 (428884.53, 929630.96)	819.67 (541.18, 1158.56)	1454389.99 (863374.75, 2234460.47)	569.17 (338.95, 872.73)	-1.22 (-1.32, -1.11)	58234.00 (33439.54, 89547.99)	72.28 (42.42, 110.02)	129268.90 (66944.42, 213695.18)	50.46 (26.30, 83.30)	-1.20 (-1.31, -1.10)
**Eastern Europe**	31361.30 (18452.01, 49750.16)	95.49 (56.12, 152.49)	25028.93 (13395.68, 42954.56)	53.66 (28.65, 92.41)	-1.99 (-2.16, -1.81)	2805.30 (1383.29, 4920.12)	8.52 (4.21, 14.99)	2249.04 (1030.78, 4138.58)	4.82 (2.20, 8.92)	-1.97 (-2.16, -1.78)
**Eastern Sub-Saharan Africa**	26019.88 (12664.77, 45977.33)	389.05 (191.97, 676.96)	44564.83 (21059.73, 81110.56)	297.04 (140.93, 537.68)	-0.86 (-0.92, -0.80)	2299.89 (1004.52, 4229.73)	34.08 (15.17, 61.42)	3946.65 (1676.98, 7640.96)	26.14 (11.22, 50.26)	-0.84 (-0.91, -0.78)
**High-income Asia Pacific**	13243.22 (6385.06, 22504.78)	60.47 (29.71, 102.75)	55667.43 (31806.16, 89561.57)	70.14 (40.46, 111.28)	0.47 (0.43, 0.51)	1195.27 (489.99, 2223.78)	5.44 (2.29, 10.12)	4982.50 (2437.97, 8919.21)	6.31 (3.08, 11.23)	0.48 (0.44, 0.52)
**High-income North America**	87158.48 (49762.18, 137296.11)	181.36 (103.90, 285.54)	278184.49 (169284.21, 440243.65)	306.53 (186.76, 484.62)	1.73 (1.66, 1.80)	7863.92 (3903.73, 13624.77)	16.37 (8.15, 28.35)	25056.91 (12935.72, 44069.73)	27.62 (14.26, 48.52)	1.73 (1.66, 1.79)
**North Africa and Middle East**	15994.92 (11160.29, 22156.78)	102.42 (70.91, 143.09)	49933.21 (33796.91, 71242.22)	113.37 (76.37, 162.64)	0.32 (0.24, 0.41)	1421.89 (799.81, 2242.75)	9.04 (5.12, 14.28)	4448.81 (2489.37, 7094.13)	10.06 (5.64, 16.12)	0.34 (0.24, 0.43)
**Oceania**	1134.05 (795.53, 1547.99)	509.58 (359.35, 698.19)	2720.24 (1901.09, 3751.41)	468.05 (326.22, 646.02)	-0.28 (-0.31, -0.25)	100.52 (57.10, 154.49)	44.75 (26.22, 67.39)	240.80 (137.33, 372.72)	41.18 (23.90, 62.89)	-0.27 (-0.30, -0.24)
**South Asia**	225702.19 (146777.69, 330806.68)	475.56 (309.08, 696.93)	665324.67 (404939.52, 1031670.99)	457.88 (277.63, 710.08)	-0.16 (-0.22, -0.10)	19557.50 (11071.00, 32201.01)	40.88 (23.26, 67.01)	57979.18 (30576.99, 99781.22)	39.69 (21.00, 68.27)	-0.12 (-0.17, -0.07)
**Southeast Asia**	61518.31 (43057.11, 85687.37)	263.95 (183.51, 369.82)	148498.77 (101355.76, 213983.06)	233.14 (158.85, 335.85)	-0.42 (-0.48, -0.36)	5444.91 (3200.80, 8553.89)	23.23 (13.70, 36.67)	13152.06 (7593.76, 21017.10)	20.56 (11.91, 32.83)	-0.41 (-0.46, -0.36)
**Southern Latin America**	4825.23 (2990.31, 7121.95)	89.55 (55.30, 132.95)	14011.39 (8388.14, 20938.21)	121.02 (72.59, 180.62)	0.99 (0.94, 1.04)	432.82 (215.93, 727.83)	8.01 (4.03, 13.51)	1254.73 (619.80, 2100.23)	10.84 (5.36, 18.15)	0.99 (0.89, 1.08)
**Southern Sub-Saharan Africa**	13316.92 (6823.32, 23185.46)	481.46 (246.44, 839.38)	23426.11 (11640.81, 40888.92)	408.89 (203.28, 714.79)	-0.53 (-0.64, -0.42)	1186.29 (539.65, 2206.86)	42.75 (19.51, 79.59)	2095.24 (928.36, 3962.29)	36.42 (16.24, 69.02)	-0.52 (-0.62, -0.41)
**Tropical Latin America**	18083.61 (11090.34, 27797.69)	207.13 (127.26, 316.03)	68266.00 (37595.45, 111318.43)	223.80 (123.60, 364.11)	0.23 (0.16, 0.30)	1598.15 (840.98, 2646.02)	18.21 (9.68, 29.88)	6043.79 (2988.29, 10640.59)	19.80 (9.82, 34.77)	0.25 (0.18, 0.31)
**Western Europe**	104458.89 (68102.71, 149872.17)	134.26 (87.91, 192.52)	282416.00 (192494.42, 397113.87)	206.88 (141.33, 289.69)	1.41 (1.32, 1.51)	9409.35 (5214.87, 14902.92)	12.09 (6.72, 19.12)	25384.34 (14548.38, 39644.49)	18.64 (10.69, 29.07)	1.41 (1.32, 1.51)
**Western Sub-Saharan Africa**	24485.02 (11773.18, 44580.70)	291.16 (140.51, 525.32)	38271.65 (17937.35, 71849.16)	216.47 (101.75, 406.14)	-0.95 (-0.98, -0.92)	2164.52 (926.67, 4143.80)	25.57 (11.07, 48.45)	3399.97 (1423.02, 6625.56)	19.13 (8.07, 37.41)	-0.93 (-0.96, -0.90)

**Abbreviation: YLDs, years lived with disability; COPD, chronic obstructive pulmonary disease; HF, heart failure; AAPC, average annual percentage change; UI, uncertainty interval; ASR, age-standardized rate; CI, confidence interval; SDI, socio-demographic index.**

The burden of COPD-HF comorbidity was also assessed among participants by sex, age, SDI, and region. These data generated for stratified analysis had high value beyond the scope of this study and were deposited into figshare, a public repository, for free and easy reuse. Consistent upward trends in both prevalent cases and YLDs were observed in either men or women, while prevalent cases and YLDs were predominant in men (Fig. 2). In terms of ASRs of COPD-HF comorbidity prevalence and YLDs, they each remained higher in men than women throughout the study period, although both of them increased in women but decreased in men (Fig. 2 and Supplementary Table 3).

The burden of COPD-HF comorbidity in older adults by age was explored (Table 2). In 1990 and 2021, the peaks of prevalent cases of COPD-HF comorbidity were each observed among those aged 70-74 years. An initially increasing and subsequently decreasing trend was found in age-specific rates of both prevalence and YLDs of COPD-HF comorbidity, with the peak in those aged 90-94 years in the year of 1990 and 2021, separately. Similar scenarios of trends in crude numbers and rates of COPD-HF comorbidity were also investigated for men and women in 2021, respectively (Fig. 3).

Among the four severity subtypes of COPD-HF comorbidity, the severe and treated subtypes were the top two greatest contributors to the prevalent cases and YLDs in 2021, which was consistently observed across sex and age, separately (Fig. 4). Similarly, the highest ASRs of COPD-HF comorbidity prevalence and YLDs were observed in treated and severe subtypes in 2021, respectively. Additionally, the maximal ASRs of prevalence and YLDs of severe/treated COPD-HF comorbidity occurred in men and those aged 90-94 years, respectively.

**Figure 2.**
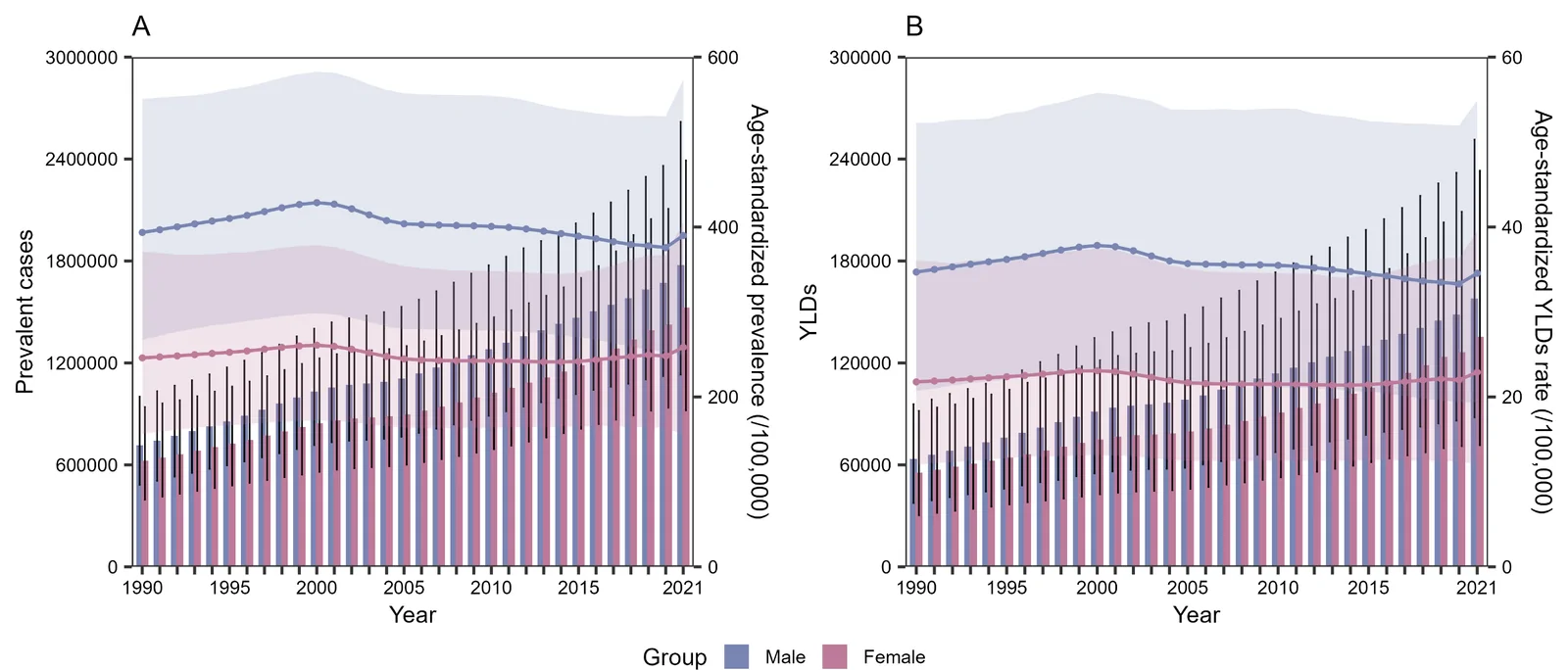
**Number and age-standardized rate of prevalence and YLDs for global COPD-HF comorbidity in adults aged 60 and older from 1990 to 2021, by sex**. (**A**) Number and age-standardized rate of prevalence; (B) Number and age-standardized rate of YLDs. YLDs, years lived with disability; COPD, chronic obstructive pulmonary disease; HF, heart failure

Based on the prediction analysis, the estimated ASR of COPD-HF comorbidity prevalence would continue to increase annually from 315.55 per 100,000 population in 2021 to 387.45 per 100,000 population in 2050 (Fig. 5A). Moreover, the ASR of YLDs would also increase annually from 27.94 per 100,000 population in 2021 to 34.73 per 100,000 population in 2050 (Fig. 5B).

**Table 2:** Number and rate of prevalence and YLDs for COPD-HF comorbidity in older adults (aged 60+ years) in 1990 and 2021 by age.

Age group	Prevalence				YLDs			
	Number (95% UI), 1990	Rate per 100,000 (95% UI), 1990	Number (95% UI), 2021	Rate per 100,000 (95% UI), 2021	Number (95% UI), 1990	Rate per 100,000 (95% UI), 1990	Number (95% UI), 2021	Rate per 100,000 (95% UI), 2021
**60 to 64 years**	98430.71 (63404.32, 140448.41)	61.29 (39.48, 87.45)	171512.76 (102474.05, 264879.83)	53.59 (32.02, 82.76)	8924.77 (4985.54, 14701.12)	5.56 (3.10, 9.15)	15570.30 (8463.09, 26320.98)	4.86 (2.64, 8.22)
**65 to 69 years**	256580.24 (165534.49, 371567.53)	207.57 (133.92, 300.60)	506503.97 (302237.84, 783556.81)	183.62 (109.57, 284.06)	23067.84 (12983.60, 36865.52)	18.66 (10.50, 29.82)	45603.07 (23626.15, 77079.19)	16.53 (8.57, 27.94)
**70 to 74 years**	322090.35 (214232.30, 460986.68)	380.45 (253.05, 544.51)	705202.50 (446005.50, 1069859.88)	342.60 (216.68, 519.76)	28723.35 (16429.61, 43828.38)	33.93 (19.41, 51.77)	63116.67 (34770.15, 102346.17)	30.66 (16.89, 49.72)
**75 to 79 years**	290393.11 (188104.92, 423042.62)	471.76 (305.59, 687.25)	662382.10 (411403.93, 1001047.30)	502.24 (311.94, 759.03)	25671.06 (14105.19, 40834.10)	41.70 (22.91, 66.34)	58815.19 (31913.70, 96785.35)	44.60 (24.20, 73.39)
**80 to 84 years**	214039.54 (143225.03, 303268.68)	605.04 (404.87, 857.27)	591264.16 (377064.05, 858636.57)	675.09 (430.52, 980.37)	18722.86 (11569.65, 28773.19)	52.93 (32.70, 81.34)	52064.10 (29218.59, 84321.15)	59.45 (33.36, 96.28)
**85 to 89 years**	116225.51 (80136.51, 165986.71)	769.14 (530.32, 1098.44)	428037.69 (267727.98, 642981.03)	936.18 (585.56, 1406.29)	10069.83 (6048.06, 15955.55)	66.64 (40.02, 105.59)	37376.62 (20883.97, 60503.55)	81.75 (45.68, 132.33)
**90 to 94 years**	34212.39 (22962.46, 49673.86)	798.39 (535.86, 1159.20)	185011.61 (118404.47, 274670.37)	1034.20 (661.87, 1535.38)	2929.40 (1787.34, 4468.58)	68.36 (41.71, 104.28)	16004.52 (9298.51, 26238.70)	89.46 (51.98, 146.67)
**95+ years**	7271.67 (4216.12, 11560.04)	714.25 (414.12, 1135.47)	53526.61 (28596.19, 89796.06)	982.08 (524.67, 1647.54)	615.02 (337.13, 1050.93)	60.41 (33.11, 103.23)	4584.04 (2331.90, 8439.20)	84.11 (42.78, 154.84)

Abbreviation: YLDs, years lived with disability; COPD, chronic obstructive pulmonary disease; HF, heart failure; UI, uncertainty interval.

**Figure 3.**
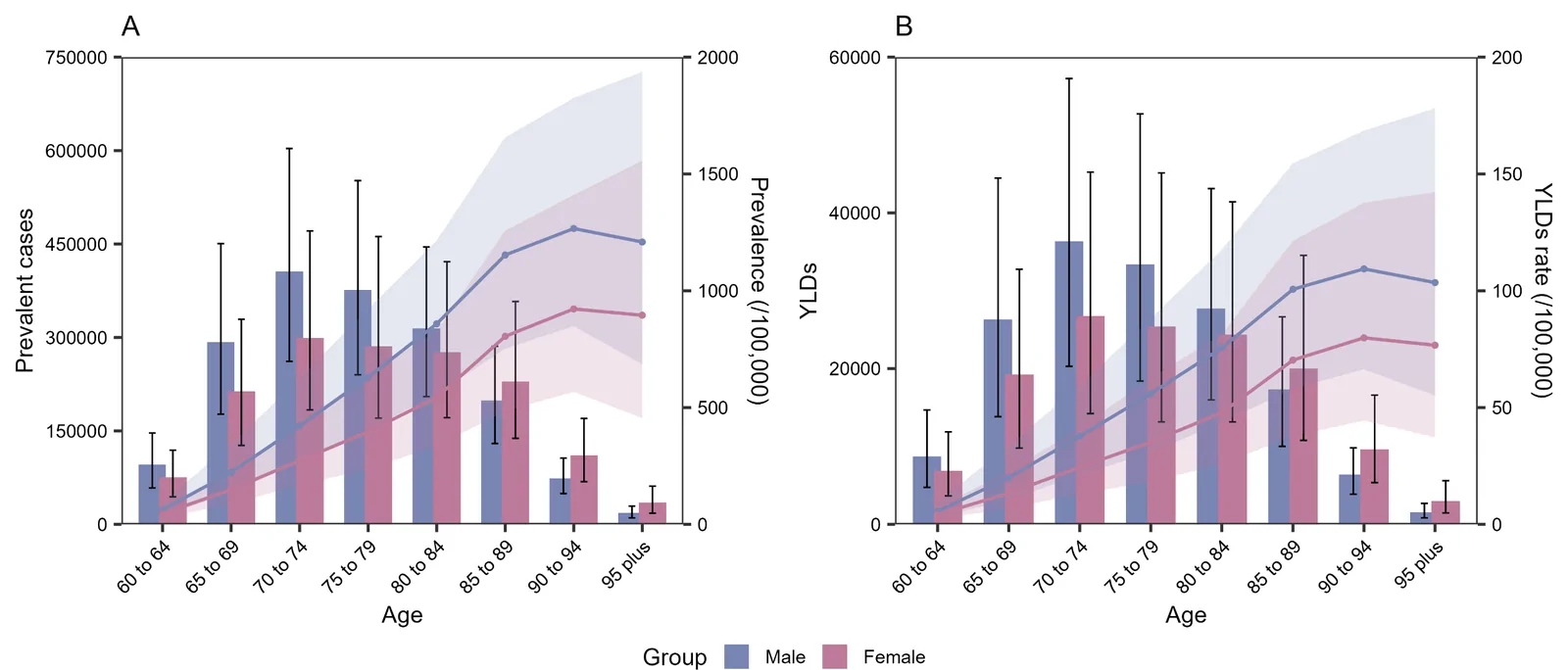
**Age-specific burden of global COPD-HF comorbidity in adults aged 60 and older in 2021, by sex**. (**A**) Number and crude rate of prevalence; (B) Number and crude rate of YLDs. COPD, chronic obstructive pulmonary disease; HF, heart failure; YLDs, years lived with disability

### Global burden of COPD-HF comorbidity in older adults from 1990 to 2021 by region

Among the five SDI regions, the middle SDI region seemed to have the highest ASR of COPD-HF comorbidity prevalence in both 1990 and 2021 (Table 1). However, an unfavorable upward trend in ASR of prevalence was observed in high SDI region from 1990 to 2021 (AAPC: 1.19; 95% CI: 1.14, 1.24). The overall decreasing trends in ASR of COPD-HF comorbidity prevalence were consistent across high-middle, middle, low-middle, and low SDI regions, although the degrees of change were different among them (Fig. 1). For YLDs, the highest ASR was examined also in middle SDI region in 2021 (Supplementary Fig. 1). YLDs and prevalence of COPD-HF comorbidity shared similar AAPC patterns of ASR, with an increase in high SDI region (AAPC: 1.19; 95% CI: 1.14, 1.24) and decreases in other SDI regions.

Among the 21 WHO geographical regions, East Asia had the highest ASR of prevalence (569.17; 95% UI: 338.95, 872.73) and YLDs (50.46; 95% UI: 26.30, 83.30) of COPD-HF comorbidity in 2021 (Table 1). In contrast, Eastern Europe had the lowest ASR of prevalence and YLDs at 53.66 (95% UI: 28.65, 92.41) and 4.82 (95% UI: 2.20, 8.92), respectively, in 2021. Moreover, from 1990 to 2021, Eastern Europe exhibited the highest decreases in ASRs of prevalence and YLDs, with AAPCs of -1.99 (95% CI: -2.16, -1.81) and -1.97 (95% CI: -2.16, -1.78), respectively, whereas North America, a high-income region, showed the most substantial increases in ASRs of prevalence and YLDs, with AAPCs of 1.73 (95% CI: 1.66, 1.80) and 1.73 (95% CI: 1.66, 1.79), respectively.

### Global burden of COPD-HF comorbidity in older adults from 1990 to 2021 by country/territory

Figure 6 and Supplementary Table 4 display the burden of COPD-HF comorbidity in older adults by country/territory from 1990 to 2021. Among the 204 countries or territories worldwide, Papua New Guinea had the highest ASR of COPD-HF comorbidity prevalence in 2021 [632.53 (95% UI: 440.85, 870.22)]. In contrast, Uzbekistan had the lowest value [21.20 (95% UI: 11.45, 33.54)]. Moreover, similar patterns were observed in ASR of YLDs in 2021, with Papua New Guinea [55.57 (95% UI: 32.11, 84.65)] ranking highest and Uzbekistan [1.92 (95% UI: 0.77, 3.54)] ranking lowest. Additionally, from 1990 to 2021, Belarus exhibited the highest decrease in ASR of prevalence (AAPC: -4.73; 95% CI: -4.99, -4.46), whereas Norway showed the most substantial increase (AAPC: 3.92; 95% CI: 3.71, 4.14). The full country/territory-level dataset was also publicly available in the repository of figshare.

### Relationship between SDI and COPD-HF comorbidity Burden

Globally and across the 21 WHO geographical regions, significant negative associations of SDI with ASRs of COPD-HF comorbidity prevalence and YLDs were identified from 1990 to 2021 (ASR of prevalence: *r* = -0.38, *p* < 0.001; ASR of YLDs: *r* = -0.37, *p* < 0.001). With SDI rising, the COPD-HF comorbidity burden decreased significantly worldwide from 1990 to 2021. East Asia showed the most substantial decline in disease burden, while Australasia exhibited the most pronounced increase (Fig. 7). Among the 204 countries/territories worldwide, a similar linear relationship was found between SDI and the ASR of prevalence and YLDs of COPD-HF comorbidity in 2021 (ASR of prevalence: *r* = -0.39, *p* < 0.001; ASR of YLDs: *r* = -0.38, *p* < 0.001) (Supplementary Fig. 2).

**Figure 4.**
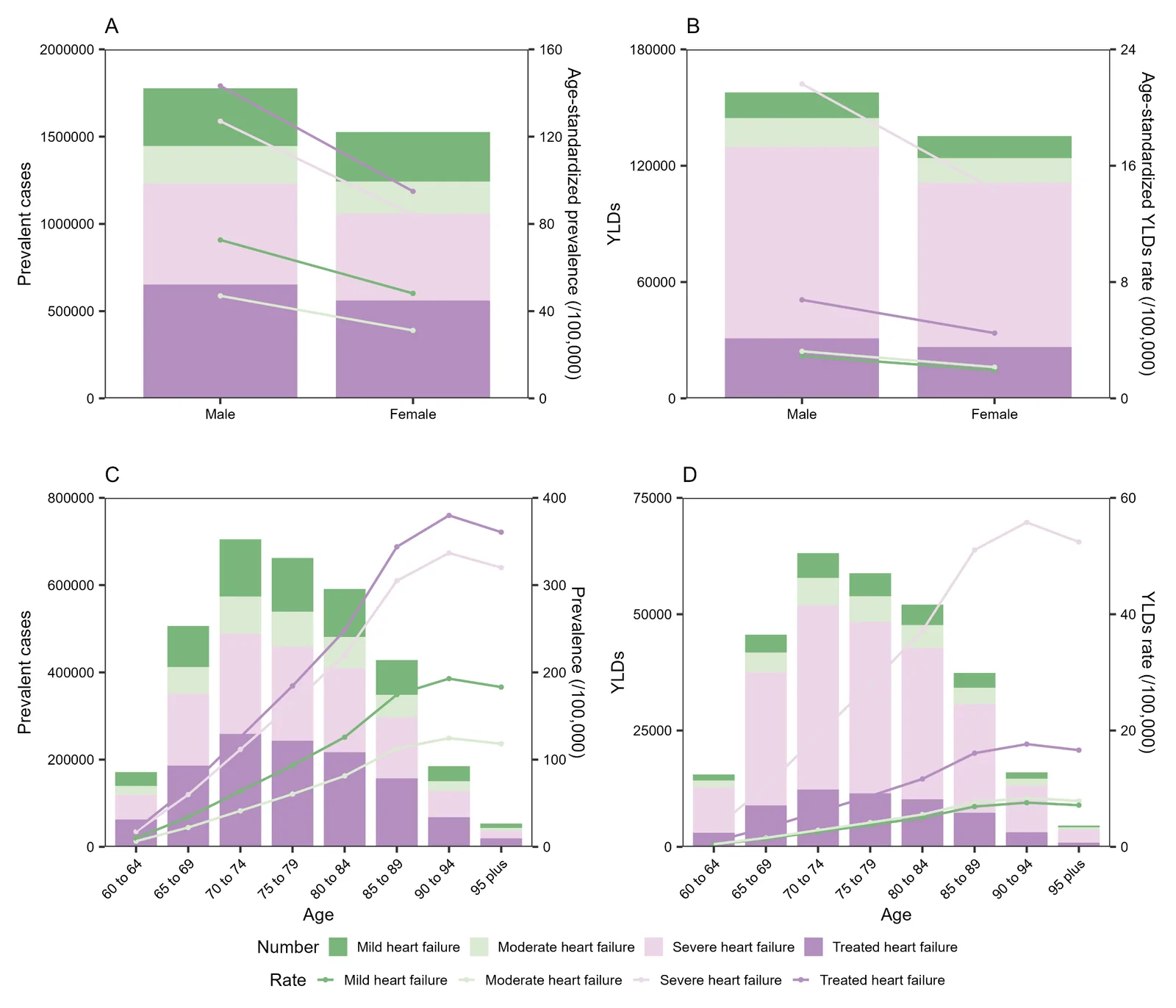
**Sex- and age-specific contribution according to the severity of global COPD-HF comorbidity in adults aged 60 and older in 2021**. (**A**) Sex-specific relative contribution to the number and age-standardized rate of prevalence, and (B) YLDs; (C) Age-specific relative contribution to the number and crude rate of prevalence, and (D) YLDs. COPD, chronic obstructive pulmonary disease; HF, heart failure; YLDs, years lived with disability

## DISCUSSION

This study, using data from GBD 2021 dataset, systematically analyzed the burden of COPD-HF comorbidity from 1990 to 2021, and made a prediction till 2050 among global adults aged 60 years or above. It was found that the global burden of COPD-HF comorbidity was substantially heavy with a consistent rising trend from 1990 to 2021, and the disease burden was projected to continue rising till 2050. The disparities of burden of COPD-HF comorbidity were observed in sex, socioeconomic development level, and geographic region worldwide.

**Figure 5.**
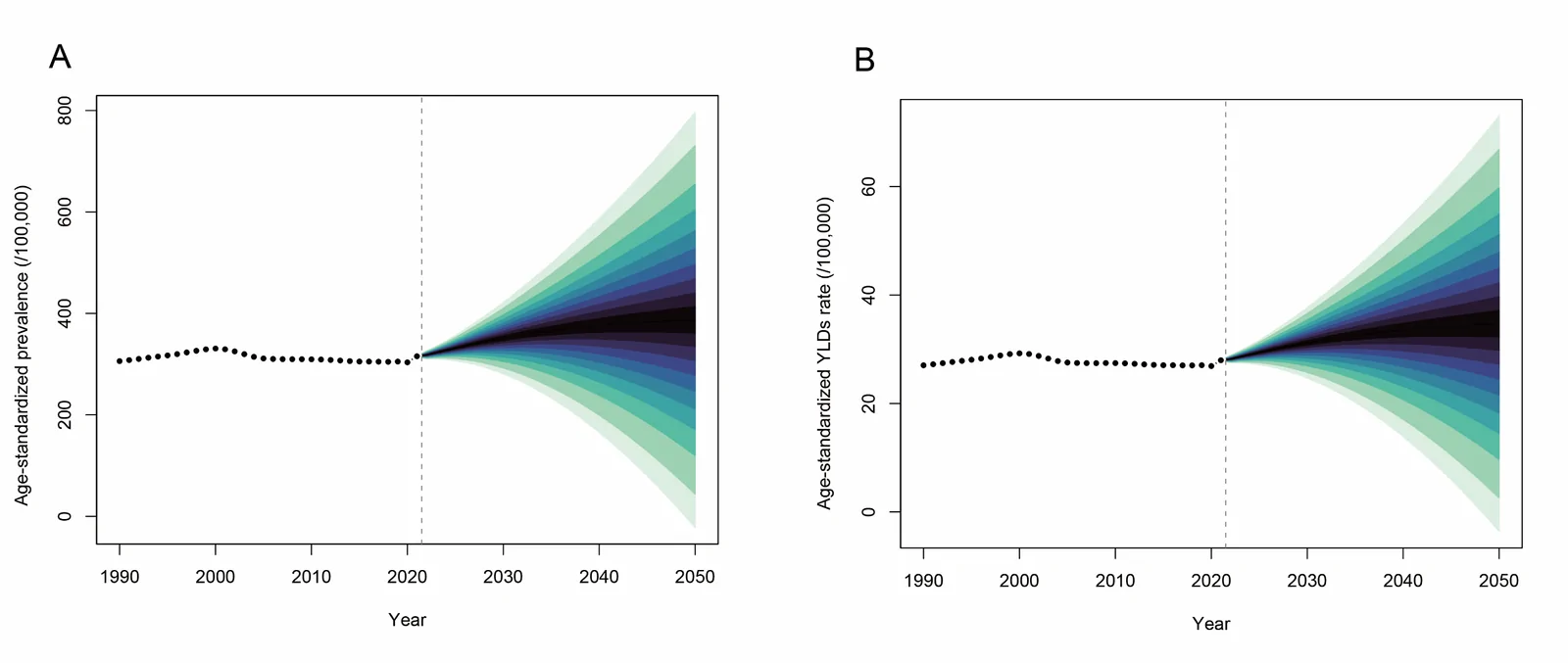
**Projection analysis in the age-standardized rates of prevalence and YLDs for global COPD-HF comorbidity in adults aged 60 and older from 1990 to 2050**. (**A**) Age-standardized rate of prevalence; (B) Age-standardized rate of YLDs. Temporal trends of COPD-HF comorbidity burden in 1990-2021 are shown by dots, and projection trends in 2022-2050 are shown by a black curve. The shaded regions represent the 95% confidence intervals of predicted values. YLDs, years lived with disability; COPD, chronic obstructive pulmonary disease; HF, heart failure

Cardiovascular diseases (CVDs) remain the major cause of death globally, with HF being its end-stage manifestation [29]. Another major cause of global mortality is respiratory diseases, including COPD. It has been well documented that COPD is associated with an increased risk of HF, likely because: 1) COPD may increase HF patient’s heart workload; 2) they share risk factors and pathophysiological mechanisms, such as adverse pulmonary vascular remodeling and chronic systemic inflammation; and 3) they act synergistically as negative prognostic factors [30]. Furthermore, as the well-known age-related diseases, both COPD and HF predominantly affect the quality of life for older adults [31]. Although both conditions impair functionality and compromise health, HF and COPD are frequently under-diagnosed and under-treated in older adults [32, 33]. This study is the first one comprehensively assessing COPD-HF comorbidity burden and its temporal trends among individuals aged 60 or above, which might provide insights into the epidemiologic patterns of this comorbidity for this vulnerable population.

**Figure 6.**
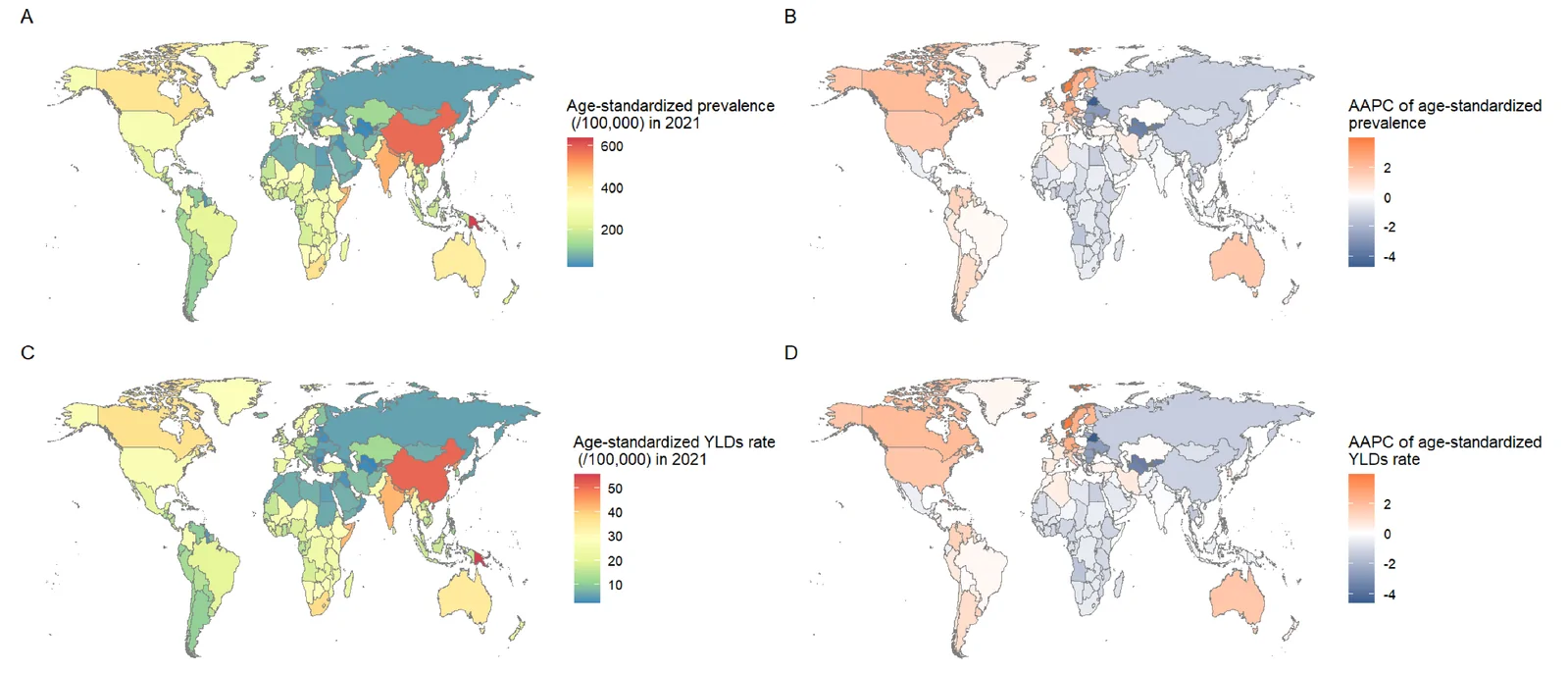
**Age-standardized rates of prevalence and YLDs in 2021, and average annual percentage changes (AAPCs) of age-standardized prevalence and YLDs rate from 1990 to 2021 for COPD-HF comorbidity in adults aged 60 and older, by country**. (**A**) Age-standardized rate of prevalence; (B) AAPC of age-standardized prevalence rate; (C) Age-standardized rate of YLDs; (D) AAPC of age-standardized YLDs rate. YLDs, years lived with disability; COPD, chronic obstructive pulmonary disease; HF, heart failure

Our findings revealed that over 3.3 million HF patients aged 60 or above progressed from COPD in 2021, representing more than double the number recorded in 1990. The number of YLDs for COPD-HF comorbidity in older adults exhibited a substantially increasing trend from 1990 to 2021 globally. Considering the influence of population growth and aging, we adjusted crude numbers to ASRs, and also found an increasing trend in ASRs for COPD-HF comorbidity among older adults from 1990 to 2021. Specifically, ASRs of both prevalence (AAPC: 0.08) and YLDs (AAPC: 0.09) increased over the study time period. Projections suggested that the increasing trend in ASRs of COPD-HF comorbidity prevalence and YLDs would continue till 2050. This rising trend might be propelled by increased life expectancy, ageing of global population, and particularly potential artificial efforts (e.g. early detection and advanced treatment) [14, 30, 34]. Moreover, as an established therapy option in clinical guidelines, β-Blockers were under-used in and under-dosed for patients with HFrEF (heart failure with reduced ejection fraction) and COPD [35, 36]. This implies the potential enormous economic burden that COPD-HF comorbidity may impose on global healthcare systems.

**Figure 7.**
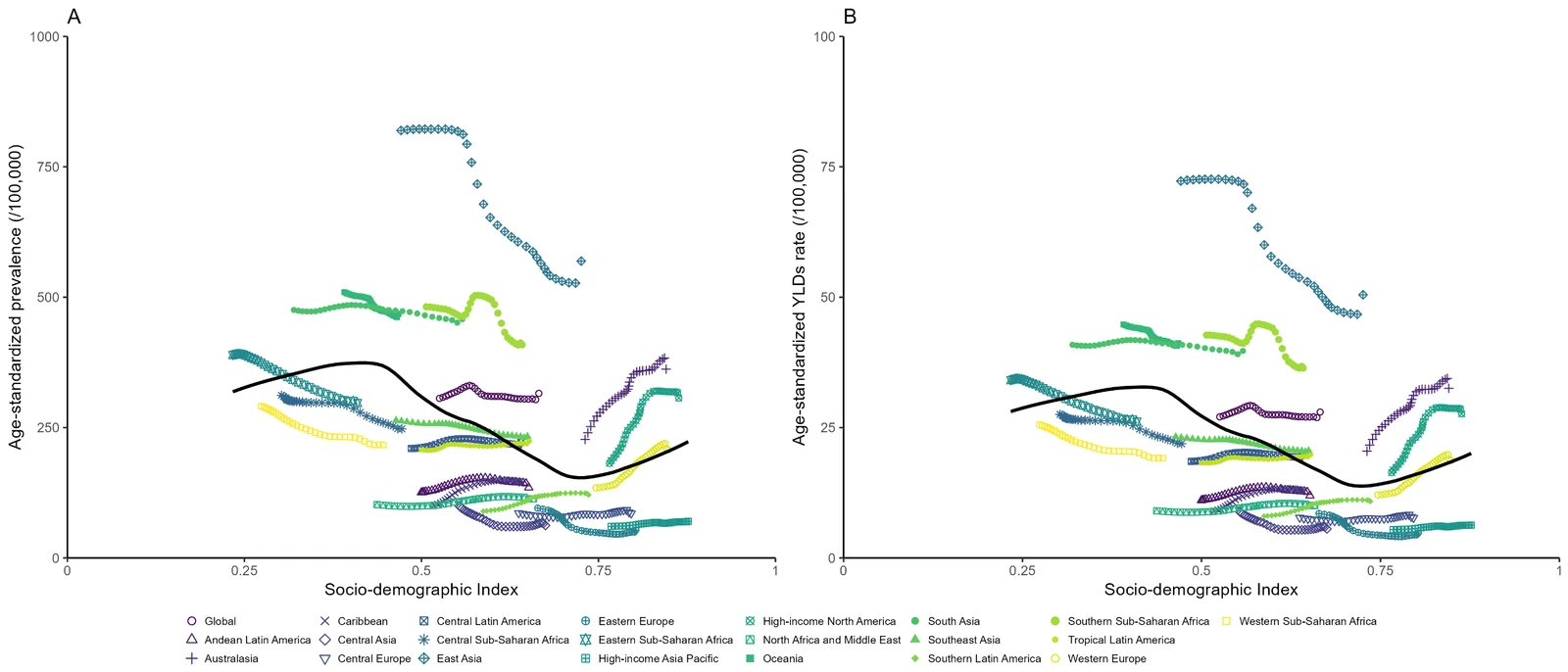
**The correlation between SDI and age-standardized rate of prevalence and YLDs for COPD-HF comorbidity in adults aged 60 and older, by globe and 21 WHO geographical regions**. (**A**) SDI and age-standardized prevalence; (B) SDI and age-standardized YLDs rate. SDI, socio-demographic index; YLDs, years lived with disability; COPD, chronic obstructive pulmonary disease; HF, heart failure

Our findings highlighted that there was a sex difference in the burden of COPD-HF comorbidity among individuals aged 60 or above. Over the past 32 years, the numbers and ASRs of both COPD-HF comorbidity prevalence and YLDs were consistently higher in men than women, which was in line with previous reports [37, 38]. This sex disparity might be caused by physiologic differences in prevalence of smoking and the protective effect of estrogen [39]. From 1990 to 2021, upward trends in the number of prevalent cases and YLDs persisted in both sexes, whereas an increase in the ASRs of prevalence and YLDs was observed only in women. This sex disparity in trends of COPD-HF comorbidity burden might be, at least partially, explained by increasing tobacco consumption among women and their greater susceptibility to the effects of smoking [40, 41]. It emphasizes the importance of considering sex disparities in the disease management of COPD-HF comorbidity. Specifically, for men, they are strongly encouraged to give up tobacco use, while, for women, they are advocated to take early screening of lung function and reduce exposure to risk factors due to their greater susceptibility. Moreover, public health policies shall integrate sex-specific strategies for prevention and management of COPD-HF comorbidity.

In terms of age-related patterns, both COPD-HF comorbidity prevalence and YLD rates exhibited unimodal distributions, peaking among adults aged 90-94 years in both 1990 and 2021. This may be explained by that both HF and COPD, as the common morbidities prevalent among older adults, are mediated by chronic inflammation and oxidative stress [42]. Additionally, aging itself is a risk factor for both COPD and HF [31]. Notably, the relationship between age and COPD-HF comorbidity burden was non-linear, which was also documented previously [43].

Among the older adults affected with COPD-HF comorbidity in 2021, although the majority of prevalent cases were attributed to treated COPD-HF comorbidity (the lowest level of severity), severe COPD-HF comorbidity (the highest level of severity) was the main contributors to YLDs worldwide. This suggests a deplorable scenario of heart and lung health for the global population if no effective actions are implemented to tackle severe COPD-HF comorbidity. Notably, the overlapping symptoms (e.g., dyspnea) between COPD and HF usually complicate diagnosis of HF in COPD patients, which frequently results in delayed diagnosis of COPD-induced HF [44, 45]. Therefore, early-diagnosis and subsequent treatment of HF in COPD patients are critically important for reducing the burden of COPD-HF comorbidity.

Regional differences were also observed in COPD-HF comorbidity among older adults worldwide in this study. Previous studies documented that populations with lower socioeconomic status suffered a heavier burden of COPD-HF comorbidity [46, 47]. Some less-developed regions, such as East Asia (primarily China), were observed to have the highest COPD-HF comorbidity burden in this study. The substantial disease burden of COPD-HF comorbidity in China might be due to several influencing factors: a large and rapidly aging population, CVDs-related unfavorable lifestyles, and high prevalence of smoking [48]. Additionally, uneven distribution of medical resources might prevent timely and effective treatment for COPD-HF patients in certain regions, thereby exacerbating the disease burden [49]. In contrast, high-SDI region (e.g., North America) has seen a slow rise in COPD-HF comorbidity burden, with a relatively low baseline data, from 1990 to 2021. Advances in diagnostic technology and increased awareness of follow-up care among COPD-HF patients might underlie this pattern in high-SDI region [47]. Further correlation analysis highlighted a negative association between SDI and ASR of prevalence or YLDs, underscoring the importance of improving socioeconomic levels to alleviate the burden of COPD-HF comorbidity. Notably, even in high-SDI European region, Norway exhibited the fastest increase in COPD-HF comorbidity burden, whereas Belarus witnessed the most significant decline. Such regional disparities aligned with previous findings [50]. Therefore, when looking at the impact of socioeconomic development on COPD-HF comorbidity burden, it might be necessary to have other factors considered, such as health-care infrastructure, accessibility of healthcare resources, age structure, and environmental factors [50].

This study has the significance worthy of mention. First, although COPD and HF, the two major chronic conditions, usually co-exist among older adults, no existing studies were available to investigate the burden of COPD-HF comorbidity. This study, using GBD data, firstly provided a comprehensive and comparable analysis of the global COPD-HF comorbidity burden among older adults from 1990 to 2021, offering important insights into the policy-making, long-term health planning and investment, and healthcare resource allocation. Second, the study revealed significant differences in COPD-HF comorbidity burden by sex and age across various populations. The older men than women bore a higher burden of COPD-HF comorbidity, while it was also age-dependent. Third, the majority burden of COPD-HF comorbidity was attributed to the severe subtype, suggesting the need for early detection and targeted management strategies to tackle COPD-HF comorbidity [36]. Additionally, COPD-HF comorbidity burden was analyzed by region. Regions with low SDI were observed to bear higher COPD-HF comorbidity burden, highlighting the unique challenges these regions face in COPD-HF comorbidity prevention and control.

The present study also has several limitations. First, the cases of COPD-HF comorbidity were defined and identified based on GBD inclusion criteria, implying that the same bias involved in GBD data might be introduced to our study. Second, due to lack of information in GBD data, another two measures of disease burden, deaths and incidence, were not analyzed in the present study. Notably, as an important measure of disease burden, the mortality of COPD-HF comorbidity could not be assessed in this study, which might lead to an under-estimation of the total disease burden and an incomplete interpretation of the comorbidity severity. Third, data on COPD-HF comorbidity were rare in some countries, so the burden estimates in GBD data were calculated primarily based on associated covariates and/or trends in neighbor countries, which might reduce the accuracy of data in these countries. Specifically, low data quality and under-report of COPD-HF comorbidity in resource-limited regions might introduce systematic bias into the estimates, thereby generating an under-estimate of COPD-HF comorbidity burden analyzed in this study. Fourth, BAPC model performed excellent in predicting disease burden using historical data with consideration of age, period, and cohort [51-53]. However, some other factors that might affect the accuracy of prediction were not involved in this study, mainly including healthcare access, treatment advancements, smoking prevalence, and aging policy interventions. This implied that the projection of COPD-HF comorbidity may be influenced by unexpected changes in these aforementioned factors, and the present prediction just displayed potential scenarios rather than definitive outcomes in future. Therefore, the findings should be interpreted with caution. The last, the ecological study design of GBD data — only population-level information involved and without details on individual patients — did not allow us to analyze individual-level differences (including SDI), risk factors and healthcare availability, potentially leading to oversight of the actual needs of high-risk individuals. Moreover, it also prevents causal inferences from the observed associations.

In conclusion, the global burden of COPD-HF comorbidity among older adults was substantially heavy with an overall rising trend from 1990 to 2021, and this increasing trend would continue till 2050. There were disparities of burden of COPD-HF comorbidity in sex, socioeconomic development level, and geographic regions worldwide. This study has important public health implications for health administrations to optimally re-allocate healthcare resources to early identification and effective treatment of COPD, HF, and particularly COPD-HF comorbidity in older adults.

## Supplementary Materials

The Supplementary data can be found online at: www.aginganddisease.org/EN/10.14336/AD.2025.1044.

## Data Availability

All the data involved in this work can be available from GBD 2021 study at http://ghdx.healthdata.org. The data on COPD-HF comorbidity among adults aged 60 years or above were obtained from the publicly available GBD 2021 dataset (https://vizhub.healthdata.org/gbd-results/). The data generated for stratified analysis were deposited into figshare for free re-use (https://doi.org/10.6084/m9.figshare.30051910.v1).

## Patient and Public Involvement

It was not appropriate or possible to involve patients or the public in the design, conduct, reporting, or dissemination plans of our research, as all data analyzed were publicly available and without personal identification.
