# Supplementary material for: Global Burden of Chronic Obstructive Pulmonary Disease and Heart Failure Comorbidity in Older Adults: An Analysis of the Global Burden of Disease Study
Source: Aging Dis. 2026 Sep 28;17(6):3500–13. doi: 10.14336/AD.2025.1044 (PMC13653710; doi:10.14336/AD.2025.1044)
Supplement: Supplementary file 1 [file AD-17-6-3500-s.pdf]

SUPPLEMENTARY DATA

Dynamic CTA-Based Whole-Brain Arterial-Venous Collateral Global Burden of Chronic Obstructive Pulmonary Disease and Heart Failure Comorbidity in Older Adults: An Analysis of the Global Burden of Disease Study

Jian Kang, Huiqing Xu, Xiaojing Deng, Yeping Bian, Tianrui Deng, Yunting Xu, Guofeng Ao, Jian Xu, Fei Xu

© 2025. Kang J et al. Published online at http://www.aginganddisease.org/EN/10.14336/AD.2025.1044

SUPPLEMENTARY DATA

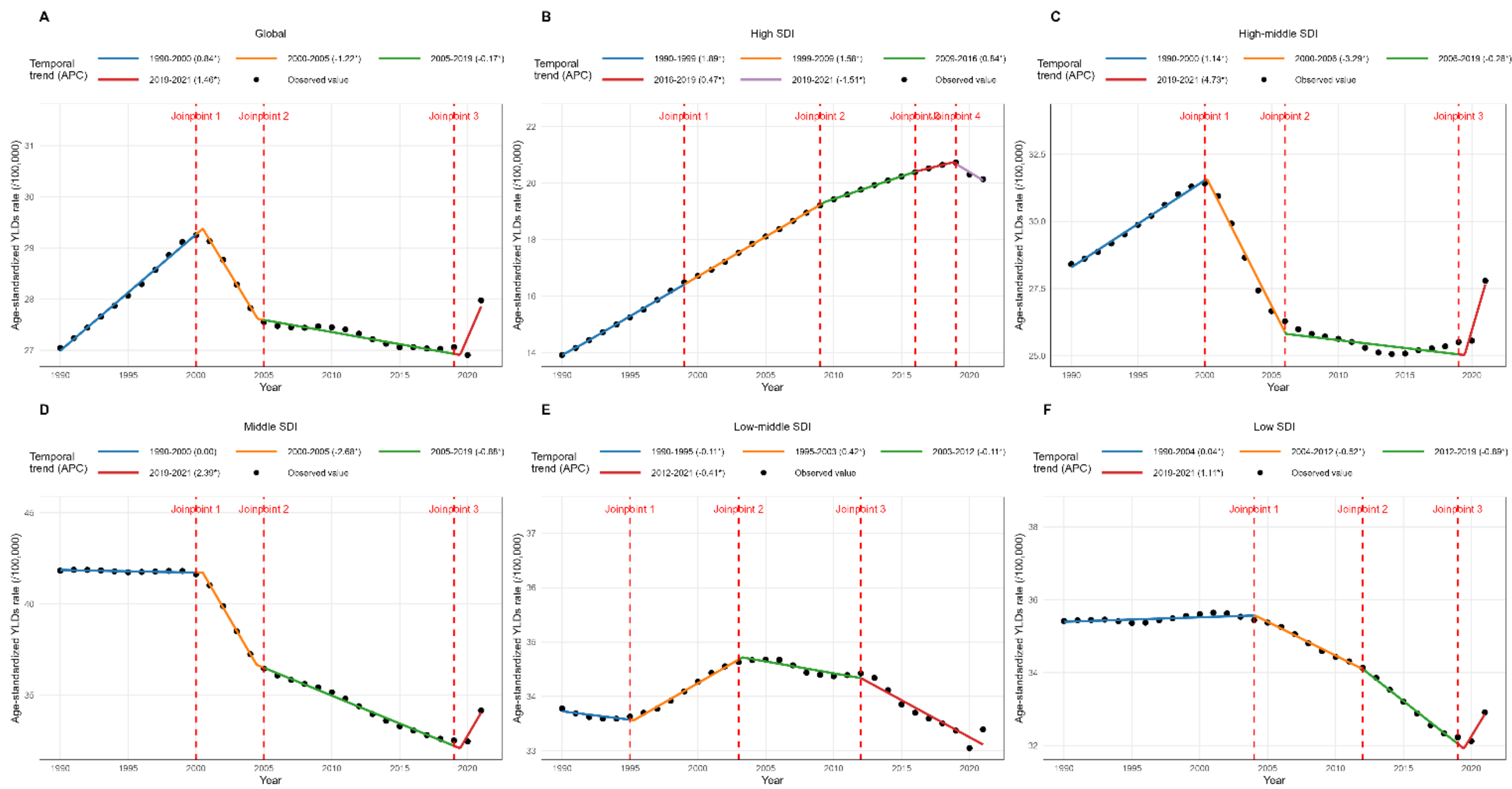

Supplementary Figure 1. Joinpoint regression analysis of the ASR of YLDs for COPD-HF comorbidity in adults aged 60 and older from 1990 to 2021 globally and by SDI quintiles. (A) Trends in the globe; (B) Trends in high SDI region; (C) Trends in high-middle SDI region; (D) Trends in middle SDI region; (E) Trends in low-middle SDI region; (F) Trends in low SDI region. * Indicates that the APC is significantly different from zero at the alpha = 0.05 level. ASR, age-standardized rate; YLDs, years lived with disability; COPD, chronic obstructive pulmonary disease; HF, heart failure; SDI, socio-demographic index; APC, annual percentage change.

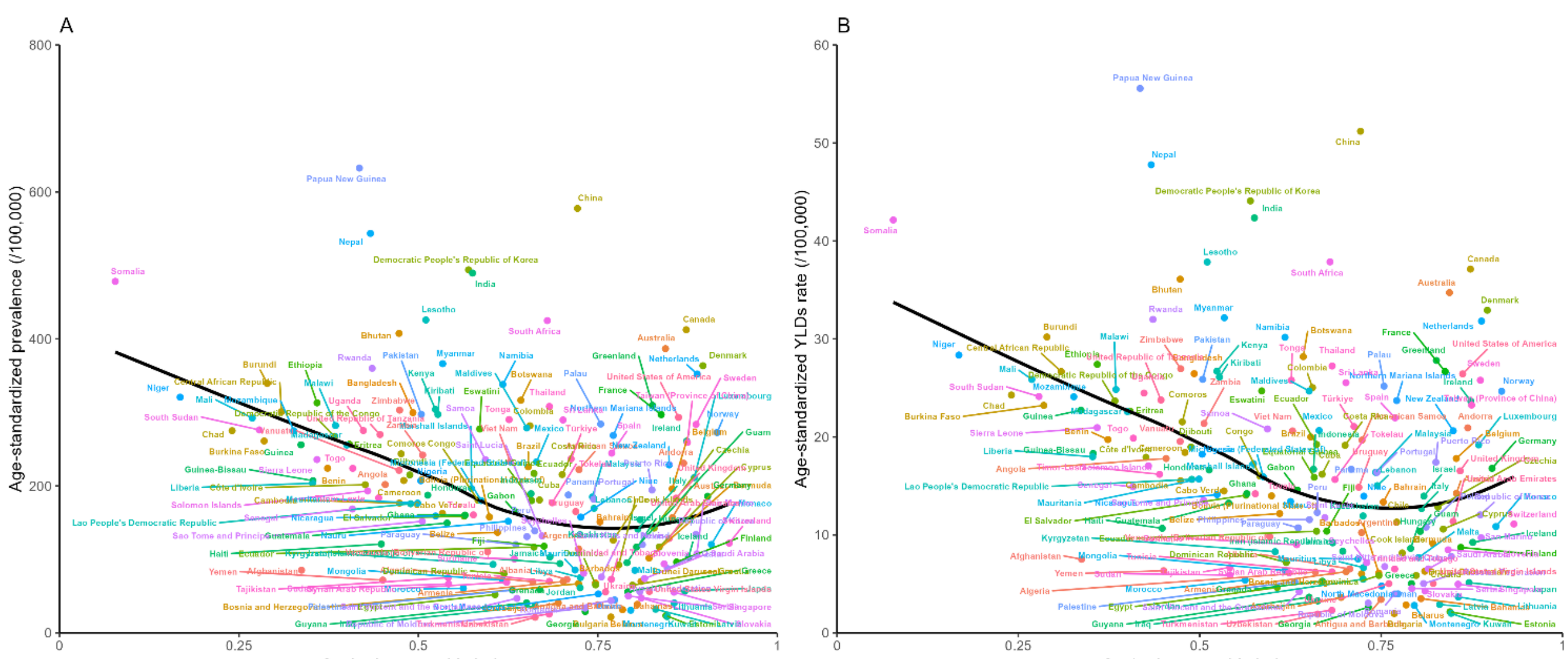

Supplementary Figure 2. The correlation between SDI and age-standardized rate of prevalence and YLDs for COPD-HF comorbidity in adults aged 60 and older, by country. (A) SDI and age-standardized prevalence; (B) SDI and age-standardized YLDs rate. SDI, socio-demographic index; YLDs, years lived with disability; COPD, chronic obstructive pulmonary disease; HF, heart failure

© 2025. Kang J et al. Published online at http://www.aginganddisease.org/EN/10.14336/AD.2025.1044

SUPPLEMENTARY DATA

Table S1. 204 countries and territories classified into each geographical region

Geographical regions	Involved countries and territories
**Central Asia**	Armenia, Azerbaijan, Georgia, Kazakhstan, Kyrgyzstan, Mongolia, Tajikistan, Turkmenistan, Uzbekistan
**Central Europe**	Albania, Bosnia and Herzegovina, Bulgaria, Croatia, Czechia, Hungary, Montenegro, North Macedonia, Poland, Romania, Serbia, Slovakia, Slovenia
**Eastern Europe**	Belarus, Estonia, Latvia, Lithuania, Republic of Moldova, Russian Federation, Ukraine
**Australasia**	Australia, New Zealand
**High-income Asia Pacific**	Brunei Darussalam, Japan, Republic of Korea, Singapore
**High-income North America**	Canada, Greenland, United States of America
**Southern Latin America**	Argentina, Chile, Uruguay
**Western Europe**	Andorra, Austria, Belgium, Cyprus, Denmark, Finland, France, Germany, Greece, Iceland, Ireland, Israel, Italy, Luxembourg, Malta, Monaco, Netherlands, Norway, Portugal, San Marino, Spain, Sweden, Switzerland, United Kingdom
**Andean Latin America**	Bolivia (Plurinational State of), Ecuador, Peru
**Caribbean**	Antigua and Barbuda, Commonwealth of the Bahamas, Barbados, Belize, Bermuda, Cuba, Dominica, Dominican Republic, Grenada, Guyana, Haiti, Jamaica, Puerto Rico, Saint Kitts and Nevis, Saint Lucia, Saint Vincent and the Grenadines, Suriname, Trinidad and Tobago, United States Virgin Islands
**Central Latin America**	Colombia, Costa Rica, El Salvador, Guatemala, Honduras, Mexico, Nicaragua, Panama, Venezuela (Bolivarian Republic of)
**Tropical Latin America**	Brazil, Paraguay
**North Africa and Middle East**	Afghanistan, Algeria, Bahrain, Egypt, Iran (Islamic Republic of), Iraq, Jordan, Kuwait, Lebanon, Libya, Morocco, Oman, Palestine, Qatar, Saudi Arabia, Sudan, Syrian Arab Republic, Tunisia, Türkiye, United Arab Emirates, Yemen
**South Asia**	Bangladesh, Bhutan, India, Nepal, Pakistan
**East Asia**	China, Democratic People's Republic of Korea, Taiwan (Province of China)
**Oceania**	American Samoa, Cook Islands, Fiji, Guam, Kiribati, Marshall Islands, Micronesia (Federated States of), Nauru, Niue, Northern Mariana Islands, Palau, Papua New Guinea, Samoa, Solomon Islands, Tokelau, Tonga, Tuvalu, Vanuatu
**Southeast Asia**	Cambodia, Indonesia, Lao People's Democratic Republic, Malaysia, Maldives, Mauritius, Myanmar, Philippines, Seychelles, Sri Lanka, Thailand, Timor-Leste, Viet Nam
**Central Sub-Saharan Africa**	Angola, Central African Republic, Congo, Democratic Republic of the Congo, Equatorial Guinea, Gabon
**Eastern Sub-Saharan Africa**	Burundi, Comoros, Djibouti, Eritrea, Ethiopia, Kenya, Madagascar, Malawi, Mozambique, Rwanda, Somalia, South Sudan, Uganda, United Republic of Tanzania, Zambia
**Southern Sub-Saharan Africa**	Botswana, Eswatini, Lesotho, Namibia, South Africa, Zimbabwe
**Western Sub-Saharan Africa**	Benin, Burkina Faso, Cabo Verde, Cameroon, Chad, Côte d'Ivoire, Gambia, Ghana, Guinea, Guinea-Bissau, Liberia, Mali, Mauritania, Niger, Nigeria, Sao Tome and Principe, Senegal, Sierra Leone, Togo

© 2025. Kang J et al. Published online at http://www.aginganddisease.org/EN/10.14336/AD.2025.1044

SUPPLEMENTARY DATA

Table S2. 204 countries and territories classified into each SDI region

SDI regions	Involved countries and territories
**Low SDI**	Afghanistan, Benin, Burkina Faso, Burundi, Central African Republic, Chad, Democratic Republic of the Congo, Eritrea, Ethiopia, Guinea, Guinea-Bissau, Haiti, Liberia, Madagascar, Malawi, Mali, Mozambique, Nepal, Niger, Papua New Guinea, Côte d'Ivoire, Gambia, Rwanda, Senegal, Sierra Leone, Solomon Islands, Somalia, South Sudan, Timor-Leste, Togo, Uganda, United Republic of Tanzania, Yemen
**Low-middle SDI**	Angola, Bangladesh, Belize, Bhutan, Venezuela (Bolivarian Republic of), Cambodia, Cameroon, Comoros, Congo, Democratic People's Republic of Korea, Djibouti, Egypt, El Salvador, Micronesia (Federated States of), Ghana, Guatemala, Honduras, India, Kenya, Eswatini, Kiribati, Kyrgyzstan, Lao People's Democratic Republic, Lesotho, Marshall Islands, Mauritania, Mongolia, Morocco, Myanmar, Namibia, Nicaragua, Nigeria, Pakistan, Bolivia (Plurinational State of), Cabo Verde, Samoa, Sao Tome and Principe, Sudan, Tajikistan, Tuvalu, Vanuatu, Zambia, Zimbabwe
**Middle SDI**	Albania, Algeria. Armenia, Azerbaijan, Botswana, Brazil, Colombia, Costa Rica, Cuba, Dominican Republic, Ecuador, Equatorial Guinea, Fiji, Gabon, Grenada, Guyana, Indonesia, Iraq, Iran (Islamic Republic of), Jamaica, Maldives, Mexico, Palestine, Panama, Paraguay, Peru, Philippines, Nauru, Saint Lucia, Saint Vincent and the Grenadines, Viet Nam, South Africa, Sri Lanka, Suriname, Syrian Arab Republic, Thailand, Tokelau, Tonga, Tunisia, Turkmenistan, Uzbekistan
**High-middle SDI**	American Samoa, Antigua and Barbuda, Argentina, Bahrain, Barbados, Belarus, Bosnia and Herzegovina, Brunei Darussalam, Bulgaria, Chile, China, Commonwealth of the Bahamas, Cook Islands, Croatia, Dominica, Georgia, Greece, Guam, Hungary, Israel, Italy, Jordan, Kazakhstan, Lebanon, Libya, Malaysia, Malta, Mauritius, Montenegro, Northern Mariana Islands, Oman, Portugal, Republic of Moldova, Niue, Palau, Romania, Russian Federation, Saint Kitts and Nevis, Serbia, Seychelles, Slovakia, Spain, North Macedonia, Trinidad and Tobago, Türkiye, Ukraine, Uruguay
**High SDI**	Andorra, Australia, Austria, Belgium, Bermuda, Canada, Cyprus, Czechia, Denmark, Estonia, Finland, France, Germany, Greenland, Iceland, Ireland, Japan, Kuwait, Latvia, Lithuania, Luxembourg, Netherlands, New Zealand, Norway, Poland, Monaco, Puerto Rico, Qatar, Republic of Korea, San Marino, Saudi Arabia, Singapore, Slovenia, Sweden, Switzerland, Taiwan (Province of China), United Arab Emirates, United Kingdom, United States of America, United States Virgin Islands

© 2025. Kang J et al. Published online at http://www.aginganddisease.org/EN/10.14336/AD.2025.1044

SUPPLEMENTARY DATA

Table S3. Age-standardized rate of prevalence and YLDs for COPD-HF comorbidity in older adults (aged 60+ years) in 1990 and 2021 and their AAPC from 1990 to 2021, by region and sex

Location	Male					Female				
	ASR per 100,000 (95% UI), 1990	ASR per 100,000 (95% UI), 2000	ASR per 100,000 (95% UI), 2010	ASR per 100,000 (95% UI), 2021	1990-2021 AAPC (95% CI)	ASR per 100,000 (95% UI), 1990	ASR per 100,000 (95% UI), 2000	ASR per 100,000 (95% UI), 2010	ASR per 100,000 (95% UI), 2021	1990-2021 AAPC (95% CI)
**Prevalence**										
Global	393.58 (267.20, 550.82)	428.71 (298.53, 582.71)	400.68 (278.15, 554.29)	389.97 (249.05, 573.13)	-0.05 (-0.09, -0.02)	245.95 (156.38, 370.73)	260.82 (172.19, 378.48)	242.49 (163.90, 347.76)	258.39 (155.89, 405.00)	0.11 (0.03, 0.20)
High SDI	211.66 (132.87, 311.54)	240.06 (156.99, 340.00)	254.03 (180.46, 340.39)	255.40 (165.02, 372.80)	0.60 (0.55, 0.65)	122.27 (77.64, 183.08)	153.57 (101.98, 221.26)	192.38 (139.16, 259.12)	203.03 (131.11, 303.01)	1.66 (1.59, 1.73)
High-middle SDI	429.24 (293.21, 597.27)	487.62 (340.99, 654.50)	417.77 (287.98, 578.13)	420.39 (264.22, 614.15)	-0.08 (-0.22, 0.06)	252.35 (159.04, 381.08)	266.87 (175.28, 388.26)	199.38 (132.15, 291.08)	234.40 (138.35, 373.57)	-0.42 (-0.64, -0.20)
Middle SDI	543.11 (375.58, 747.75)	565.70 (399.63, 759.53)	506.79 (346.44, 698.91)	476.43 (300.69, 696.54)	-0.44 (-0.52, -0.36)	415.94 (261.69, 622.48)	392.87 (259.46, 573.67)	307.48 (201.17, 453.32)	312.44 (181.68, 499.75)	-0.97 (-1.02, -0.91)
Low-middle SDI	477.04 (317.89, 684.20)	486.03 (335.41, 685.46)	474.62 (327.32, 668.31)	454.26 (289.62, 674.28)	-0.17 (-0.26, -0.09)	307.29 (196.90, 460.95)	310.93 (206.83, 454.34)	326.83 (217.91, 476.94)	324.31 (196.49, 501.84)	0.15 (0.09, 0.20)
Low SDI	415.74 (253.52, 652.56)	426.02 (268.78, 653.51)	409.72 (258.57, 628.28)	390.55 (228.13, 627.65)	-0.22 (-0.26, -0.18)	404.03 (231.22, 651.14)	396.83 (232.66, 629.24)	384.24 (229.08, 610.44)	366.66 (202.96, 612.52)	-0.33 (-0.36, -0.30)
Andean Latin America	163.95 (113.78, 228.92)	180.03 (125.91, 249.18)	190.95 (132.53, 266.85)	169.53 (112.32, 244.86)	0.15 (0.09, 0.20)	92.14 (63.03, 130.26)	104.62 (72.23, 147.33)	120.97 (83.29, 169.19)	106.38 (70.87, 154.44)	0.50 (0.46, 0.55)
Australasia	333.53 (218.08, 475.60)	382.25 (260.51, 528.39)	387.52 (272.09, 524.14)	385.82 (270.45, 532.72)	0.48 (0.44, 0.52)	164.07 (109.50, 236.81)	259.84 (175.59, 369.06)	339.79 (250.05, 446.25)	346.03 (251.98, 460.52)	2.52 (2.33, 2.71)
Caribbean	139.47 (96.12, 194.66)	183.07 (127.65, 250.90)	201.03 (140.96, 276.96)	197.90 (134.03, 278.93)	1.13 (1.09, 1.17)	67.33 (45.42, 96.98)	89.72 (61.72, 123.80)	105.71 (72.97, 147.90)	100.71 (65.85, 146.10)	1.35 (1.28, 1.42)
Central Asia	132.82 (72.71, 205.79)	108.26 (61.58, 167.13)	83.26 (46.39, 126.93)	80.11 (41.63, 127.62)	-1.56 (-1.78, -1.34)	81.88 (53.56, 120.18)	59.35 (38.51, 88.00)	45.45 (29.36, 67.24)	50.29 (30.54, 77.10)	-1.57 (-1.69, -1.44)
Central Europe	148.82 (91.63, 226.57)	134.66 (87.69, 195.22)	132.35 (92.08, 180.71)	123.81 (78.48, 183.67)	-0.60 (-0.64, -0.56)	46.93 (29.37, 71.38)	44.59 (29.52, 64.08)	53.70 (38.02, 72.98)	61.07 (39.01, 90.94)	0.83 (0.73, 0.93)
Central Latin America	270.21 (183.46, 381.05)	290.74 (200.73, 405.12)	280.50 (192.98, 396.05)	274.45 (178.26, 405.27)	0.03 (-0.02, 0.09)	155.61 (102.41, 233.26)	170.13 (115.22, 248.28)	181.26 (125.21, 258.37)	180.19 (116.47, 269.54)	0.46 (0.40, 0.52)
Central Sub-Saharan Africa	256.21 (113.92, 488.26)	241.58 (107.16, 463.49)	230.39 (102.93, 439.96)	186.30 (83.14, 359.82)	-1.03 (-1.09, -0.98)	361.28 (169.59, 667.29)	342.31 (160.74, 627.76)	337.93 (156.85, 632.36)	286.38 (131.12, 544.43)	-0.76 (-0.82, -0.69)
East Asia	871.67 (591.58, 1216.45)	916.54 (638.84, 1238.73)	751.55 (508.74, 1041.03)	688.03 (425.03, 1009.00)	-0.79 (-0.86, -0.72)	756.55 (472.09, 1132.88)	707.17 (460.76, 1028.95)	463.06 (300.62, 687.68)	465.79 (265.46, 753.58)	-1.65 (-1.80, -1.50)
Eastern Europe	166.34 (96.20, 265.24)	136.64 (81.31, 212.82)	95.33 (56.18, 147.54)	101.36 (53.62, 174.67)	-1.69 (-1.93, -1.46)	68.08 (39.78, 111.21)	42.51 (26.16, 65.89)	26.16 (16.02, 40.57)	31.05 (16.43, 54.21)	-2.64 (-2.87, -2.40)
Eastern Sub-Saharan Africa	297.65 (138.14, 550.88)	297.53 (137.28, 547.65)	255.76 (117.31, 475.74)	222.22 (102.56, 415.41)	-0.92 (-0.99, -0.84)	476.64 (242.11, 807.03)	457.49 (230.46, 775.40)	405.07 (200.92, 705.77)	357.65 (171.30, 642.37)	-0.92 (-0.97, -0.87)
High-income Asia Pacific	92.58 (42.70, 158.91)	105.31 (55.46, 170.35)	109.89 (67.01, 163.72)	113.29 (64.07, 180.93)	0.65 (0.62, 0.68)	42.40 (22.14, 71.12)	44.39 (25.06, 70.84)	42.26 (26.78, 62.31)	42.85 (24.62, 69.20)	0.04 (0.01, 0.06)
High-income North America	210.88 (116.43, 336.84)	270.19 (159.14, 414.24)	316.98 (212.18, 448.93)	294.67 (174.10, 465.60)	1.13 (0.99, 1.27)	166.24 (97.43, 260.31)	241.39 (152.58, 360.50)	325.15 (225.30, 455.86)	317.65 (193.20, 500.83)	2.15 (2.04, 2.27)

© 2025. Kang J et al. Published online at http://www.aginganddisease.org/EN/10.14336/AD.2025.1044

North Africa and Middle East	139.43 (96.14, 194.31)	134.24 (93.73, 185.95)	151.22 (115.14, 196.16)	150.26 (100.59, 216.57)	0.24 (0.15, 0.34)	67.30 (46.13, 95.68)	68.88 (47.83, 95.85)	77.96 (60.15, 99.80)	77.33 (51.71, 111.14)	0.44 (0.39, 0.50)
Oceania	619.32 (434.75, 849.92)	591.89 (410.48, 811.12)	556.94 (388.49, 764.42)	553.02 (386.52, 763.11)	-0.37 (-0.43, -0.32)	405.39 (277.34, 568.22)	407.21 (283.78, 570.77)	396.83 (272.74, 557.98)	383.50 (248.99, 565.93)	-0.17 (-0.20, -0.15)
South Asia	577.59 (380.21, 840.40)	589.12 (402.55, 837.50)	567.34 (387.69, 808.74)	539.94 (333.50, 820.20)	-0.23 (-0.28, -0.18)	368.72 (233.26, 556.01)	375.12 (245.78, 553.67)	390.62 (255.48, 577.04)	386.14 (225.36, 614.30)	0.11 (0.05, 0.18)
Southeast Asia	396.30 (273.41, 555.33)	387.07 (273.19, 534.42)	373.19 (262.22, 520.51)	357.20 (243.02, 510.59)	-0.35 (-0.40, -0.30)	164.80 (114.33, 233.28)	160.72 (113.39, 224.13)	153.40 (107.00, 214.51)	145.69 (98.37, 213.50)	-0.41 (-0.46, -0.36)
Southern Latin America	138.59 (83.89, 208.31)	158.40 (94.39, 234.18)	174.25 (104.09, 260.68)	164.78 (97.43, 247.97)	0.57 (0.50, 0.64)	56.99 (35.87, 84.83)	71.66 (43.31, 107.21)	86.01 (51.53, 128.19)	93.07 (55.72, 140.61)	1.61 (1.53, 1.69)
Southern Sub-Saharan Africa	285.90 (135.10, 523.67)	262.06 (124.73, 478.42)	286.36 (134.77, 523.59)	225.99 (105.86, 416.64)	-0.72 (-0.81, -0.62)	604.24 (312.38, 1041.57)	576.49 (294.27, 980.86)	611.66 (316.51, 1035.92)	511.09 (257.58, 885.05)	-0.54 (-0.65, -0.43)
Tropical Latin America	279.71 (175.12, 418.61)	293.33 (189.21, 425.04)	280.99 (180.43, 409.48)	283.57 (161.11, 447.57)	0.02 (-0.05, 0.09)	152.86 (90.31, 245.61)	162.56 (100.56, 251.33)	167.13 (103.49, 257.20)	183.79 (98.25, 312.89)	0.57 (0.50, 0.65)
Western Europe	211.72 (136.36, 302.84)	234.46 (160.69, 319.67)	248.46 (183.26, 323.73)	251.90 (171.35, 353.85)	0.52 (0.41, 0.63)	91.70 (60.75, 132.15)	113.78 (79.35, 158.88)	158.27 (117.65, 207.27)	175.61 (119.96, 248.92)	2.11 (2.01, 2.21)
Western Sub-Saharan Africa	250.03 (116.79, 468.44)	232.21 (107.91, 430.06)	205.16 (94.46, 386.68)	190.46 (87.40, 357.45)	-0.87 (-0.89, -0.85)	325.70 (160.07, 581.29)	293.01 (142.98, 525.69)	259.64 (123.65, 477.77)	239.28 (113.18, 446.61)	-0.99 (-1.01, -0.96)
**YLDs**										
Global	34.70 (20.72, 52.26)	37.83 (22.98, 55.80)	35.49 (21.12, 53.96)	34.55 (19.36, 54.86)	-0.04 (-0.07, 0.00)	21.77 (12.00, 36.04)	23.09 (13.17, 37.46)	21.50 (12.49, 34.56)	22.91 (12.12, 39.48)	0.12 (0.03, 0.21)
High SDI	18.98 (10.28, 30.34)	21.54 (12.23, 33.79)	22.82 (13.66, 34.77)	22.92 (12.56, 36.84)	0.60 (0.55, 0.65)	11.03 (5.98, 18.53)	13.85 (7.74, 22.56)	17.39 (10.48, 26.99)	18.31 (10.08, 30.39)	1.66 (1.59, 1.73)
High-middle SDI	38.04 (22.70, 56.15)	43.23 (26.73, 61.87)	37.21 (21.98, 56.18)	37.41 (20.49, 58.55)	-0.08 (-0.25, 0.10)	22.38 (12.24, 37.42)	23.68 (13.25, 38.36)	17.74 (10.08, 28.83)	20.84 (10.62, 36.39)	-0.41 (-0.64, -0.18)
Middle SDI	47.79 (29.22, 69.75)	49.79 (31.10, 70.93)	44.80 (26.47, 66.65)	42.10 (23.49, 65.84)	-0.43 (-0.50, -0.35)	36.70 (20.15, 60.51)	34.64 (19.67, 56.08)	27.14 (15.28, 44.46)	27.60 (14.07, 48.58)	-0.96 (-1.03, -0.90)
Low-middle SDI	41.30 (24.07, 65.58)	42.19 (24.97, 66.51)	41.30 (24.46, 64.73)	39.64 (21.87, 65.05)	-0.14 (-0.19, -0.10)	26.55 (14.87, 44.04)	26.87 (15.48, 43.48)	28.29 (16.31, 45.81)	28.16 (14.83, 48.50)	0.17 (0.11, 0.22)
Low SDI	36.04 (19.15, 62.15)	36.97 (20.36, 62.01)	35.61 (19.38, 60.13)	34.05 (17.47, 60.02)	-0.20 (-0.23, -0.16)	35.06 (17.62, 59.88)	34.44 (18.07, 58.60)	33.42 (17.55, 56.93)	31.94 (15.73, 57.25)	-0.32 (-0.37, -0.27)
Andean Latin America	14.38 (8.08, 22.58)	15.83 (8.81, 24.98)	16.87 (9.38, 26.50)	14.99 (8.14, 24.38)	0.18 (0.08, 0.27)	8.15 (4.30, 13.48)	9.27 (4.88, 15.64)	10.73 (5.71, 17.54)	9.45 (5.01, 15.92)	0.51 (0.42, 0.60)
Australasia	29.87 (15.35, 50.16)	34.40 (18.26, 55.54)	34.83 (19.13, 54.52)	34.42 (18.86, 52.99)	0.47 (0.31, 0.64)	14.87 (7.20, 25.16)	23.51 (12.07, 38.78)	30.84 (17.61, 48.33)	31.28 (17.68, 48.39)	2.47 (2.28, 2.66)
Caribbean	12.42 (6.92, 19.79)	16.26 (9.24, 25.23)	17.85 (10.01, 28.03)	17.62 (9.82, 27.93)	1.13 (1.06, 1.21)	6.03 (3.14, 10.12)	8.04 (4.20, 13.11)	9.45 (5.04, 15.38)	9.03 (4.66, 15.17)	1.35 (1.26, 1.44)
Central Asia	11.86 (5.48, 20.38)	9.65 (4.51, 16.65)	7.45 (3.41, 12.62)	7.16 (3.10, 12.63)	-1.54 (-1.77, -1.32)	7.26 (3.81, 11.87)	5.28 (2.72, 8.84)	4.06 (2.09, 6.76)	4.50 (2.22, 7.76)	-1.54 (-1.72, -1.37)
Central Europe	13.35 (6.90, 22.37)	12.11 (6.62, 19.58)	11.88 (6.86, 18.50)	11.12 (5.83, 18.39)	-0.59 (-0.65, -0.52)	4.21 (2.16, 7.18)	4.01 (2.18, 6.55)	4.84 (2.79, 7.61)	5.51 (2.98, 9.21)	0.85 (0.77, 0.93)

© 2025. Kang J et al. Published online at http://www.aginganddisease.org/EN/10.14336/AD.2025.1044

Central Latin America	23.75 (13.99, 35.60)	25.67 (15.03, 39.22)	24.79 (14.45, 38.88)	24.31 (13.53, 39.31)	0.06 (0.00, 0.12)	13.78 (7.73, 22.86)	15.13 (8.60, 24.77)	16.10 (9.41, 25.66)	16.02 (8.77, 26.85)	0.47 (0.41, 0.54)
Central Sub-Saharan Africa	22.56 (8.56, 45.60)	21.36 (7.76, 44.04)	20.34 (7.72, 42.03)	16.46 (6.05, 33.85)	-1.02 (-1.12, -0.91)	31.96 (13.04, 63.43)	30.30 (11.97, 59.83)	29.94 (12.14, 59.67)	25.38 (9.94, 52.06)	-0.75 (-0.80, -0.70)
East Asia	76.75 (47.71, 110.57)	80.83 (50.82, 113.51)	66.67 (39.07, 98.74)	61.01 (33.28, 94.93)	-0.77 (-0.83, -0.70)	66.78 (36.85, 110.09)	62.44 (35.13, 100.89)	41.01 (22.71, 67.66)	41.27 (20.54, 73.18)	-1.64 (-1.79, -1.48)
Eastern Europe	14.82 (7.15, 26.06)	12.20 (6.24, 21.00)	8.51 (4.22, 14.60)	9.07 (4.05, 16.73)	-1.68 (-1.95, -1.40)	6.08 (2.94, 11.00)	3.80 (1.96, 6.60)	2.36 (1.23, 4.08)	2.80 (1.26, 5.32)	-2.61 (-2.85, -2.37)
Eastern Sub-Saharan Africa	26.16 (10.76, 51.47)	26.17 (10.87, 51.17)	22.51 (9.29, 43.69)	19.60 (8.05, 39.00)	-0.90 (-0.99, -0.82)	41.70 (18.95, 72.60)	40.09 (18.14, 70.51)	35.63 (15.73, 64.85)	31.45 (13.74, 59.59)	-0.91 (-0.95, -0.88)
High-income Asia Pacific	8.29 (3.33, 15.65)	9.44 (4.26, 17.16)	9.87 (5.05, 16.62)	10.16 (4.83, 18.25)	0.66 (0.61, 0.71)	3.83 (1.70, 7.09)	4.00 (1.93, 7.16)	3.81 (2.00, 6.40)	3.86 (1.86, 6.90)	0.03 (-0.03, 0.10)
High-income North America	18.98 (9.11, 33.08)	24.27 (12.24, 41.17)	28.47 (16.37, 44.85)	26.51 (13.33, 46.37)	1.12 (0.98, 1.27)	15.03 (7.58, 26.07)	21.82 (11.60, 36.42)	29.37 (17.11, 46.42)	28.65 (14.85, 50.13)	2.14 (2.03, 2.26)
North Africa and Middle East	12.30 (6.94, 19.41)	11.87 (6.77, 18.89)	13.44 (8.23, 20.58)	13.33 (7.31, 21.46)	0.25 (0.15, 0.35)	5.95 (3.25, 9.74)	6.11 (3.32, 10.00)	6.90 (4.04, 10.77)	6.86 (3.70, 11.33)	0.46 (0.39, 0.54)
Oceania	54.32 (32.22, 81.54)	51.93 (30.24, 77.43)	48.93 (28.29, 73.37)	48.67 (27.47, 73.83)	-0.36 (-0.42, -0.31)	35.64 (19.55, 56.36)	35.65 (19.66, 57.19)	34.78 (19.45, 54.74)	33.72 (18.15, 55.67)	-0.18 (-0.20, -0.15)
South Asia	49.78 (28.52, 80.14)	50.89 (29.88, 80.43)	49.14 (28.73, 78.18)	46.94 (25.12, 78.87)	-0.19 (-0.25, -0.14)	31.55 (17.39, 53.31)	32.15 (18.27, 52.43)	33.59 (19.15, 54.89)	33.34 (17.28, 58.59)	0.14 (0.08, 0.21)
Southeast Asia	34.93 (20.48, 55.28)	34.09 (20.13, 52.99)	33.00 (19.56, 51.98)	31.54 (18.19, 50.26)	-0.34 (-0.38, -0.29)	14.44 (8.38, 23.02)	14.10 (8.31, 22.24)	13.48 (7.83, 21.44)	12.82 (7.26, 20.89)	-0.39 (-0.44, -0.35)
Southern Latin America	12.36 (5.92, 21.41)	14.21 (6.87, 24.23)	15.61 (7.50, 26.61)	14.73 (6.98, 24.91)	0.58 (0.50, 0.67)	5.11 (2.29, 9.27)	6.40 (2.94, 11.33)	7.73 (3.45, 13.71)	8.35 (3.84, 14.71)	1.62 (1.52, 1.73)
Southern Sub-Saharan Africa	25.34 (10.71, 50.16)	23.26 (9.64, 45.92)	25.52 (10.50, 50.40)	20.09 (8.31, 39.94)	-0.71 (-0.80, -0.61)	53.71 (24.65, 98.50)	51.27 (23.36, 93.49)	54.56 (24.59, 99.52)	45.57 (20.56, 85.48)	-0.53 (-0.64, -0.42)
Tropical Latin America	24.49 (13.35, 39.16)	25.85 (14.52, 40.33)	24.80 (14.04, 38.92)	25.00 (12.71, 41.95)	0.04 (-0.03, 0.11)	13.49 (6.82, 23.70)	14.39 (7.68, 24.62)	14.83 (7.90, 25.16)	16.31 (7.60, 30.35)	0.58 (0.51, 0.66)
Western Europe	18.99 (10.35, 29.69)	21.08 (12.07, 32.47)	22.37 (13.56, 33.69)	22.63 (12.87, 35.08)	0.54 (0.40, 0.67)	8.28 (4.58, 13.51)	10.28 (5.97, 16.32)	14.33 (8.63, 21.97)	15.87 (9.05, 25.47)	2.12 (2.01, 2.22)
Western Sub-Saharan Africa	21.98 (9.05, 43.63)	20.45 (8.54, 40.53)	18.10 (7.43, 35.70)	16.86 (6.88, 33.35)	-0.85 (-0.90, -0.81)	28.60 (12.60, 53.08)	25.71 (11.20, 48.43)	22.86 (9.74, 44.03)	21.12 (8.99, 40.84)	-0.97 (-1.00, -0.94)

Abbreviation: YLDs, years lived with disability; COPD, chronic obstructive pulmonary disease; HF, heart failure; AAPC, average annual percentage change; UI, uncertainty interval; ASR, age-standardized rate; CI, confidence interval; SDI, socio-demographic index.

© 2025. Kang J et al. Published online at http://www.aginganddisease.org/EN/10.14336/AD.2025.1044

SUPPLEMENTARY DATA

Table S4. The top and bottom ten countries or territories ranked by ASR for both prevalence and YLDs for COPD-HF comorbidity in older adults (aged 60+ years) in 2021 and their AAPC from 1990 to 2021, by sex

Characteristics	Both				Male				Female			
	Location	ASR per 100,000 (95% UI), 2021	Location	1990-2021 AAPC (95% CI)	Location	ASR per 100,000 (95% UI), 2021	Location	1990-2021 AAPC (95% CI)	Location	ASR per 100,000 (95% UI), 2021	Location	1990-2021 AAPC (95% CI)
**Prevalence**												
The top ten countries or territories ranked by ASR or AAPC	Papua New Guinea	632.53 (440.85, 870.22)	Norway	3.92 (3.71, 4.14)	China	415.86 (244.82, 628.64)	Norway	3.56 (3.42, 3.69)	Somalia	344.49 (159.90, 625.16)	Czechia	4.51 (3.92, 5.09)
	China	577.63 (341.35, 888.75)	Denmark	3.62 (3.52, 3.73)	Papua New Guinea	397.92 (268.76, 559.32)	Israel	2.98 (2.91, 3.05)	South Africa	335.90 (168.31, 581.07)	Germany	4.49 (4.32, 4.66)
	Nepal	543.64 (364.53, 781.56)	Czechia	2.94 (2.45, 3.44)	Democratic People's Republic of Korea	396.99 (261.74, 567.34)	Denmark	2.66 (2.52, 2.80)	Lesotho	308.16 (146.28, 575.48)	Norway	3.89 (3.48, 4.30)
	Democratic People's Republic of Korea	494.05 (335.57, 705.25)	Israel	2.56 (2.46, 2.67)	Nepal	347.54 (221.28, 516.09)	Czechia	2.30 (1.82, 2.79)	Papua New Guinea	306.92 (194.24, 454.85)	Netherlands	3.71 (3.45, 3.97)
	India	489.61 (287.80, 772.81)	Netherlands	2.54 (2.31, 2.77)	India	303.92 (178.71, 480.83)	Cuba	1.87 (1.84, 1.90)	Canada	302.81 (200.26, 431.62)	Finland	3.32 (3.04, 3.59)
	Somalia	478.38 (224.95, 859.98)	Germany	2.42 (2.11, 2.73)	Myanmar	284.14 (186.30, 409.16)	Finland	1.66 (1.32, 2.00)	Nepal	284.31 (187.97, 407.99)	Denmark	3.26 (3.07, 3.46)
	Lesotho	425.84 (200.73, 788.32)	Sweden	2.36 (2.26, 2.45)	Thailand	275.57 (180.02, 388.87)	Netherlands	1.54 (1.34, 1.73)	Australia	268.19 (194.43, 354.37)	Switzerland	3.23 (3.00, 3.46)
	South Africa	424.95 (212.75, 739.46)	Finland	2.17 (1.75, 2.60)	Australia	266.70 (187.52, 363.98)	Luxembourg	1.47 (1.19, 1.75)	Rwanda	260.49 (121.27, 492.06)	Israel	3.13 (3.00, 3.26)
	Canada	412.53 (276.16, 584.18)	Canada	2.07 (1.84, 2.29)	France	240.39 (158.83, 344.93)	Germany	1.47 (1.24, 1.69)	Burundi	258.60 (117.21, 491.06)	Austria	2.91 (2.61, 3.22)
	Bhutan	407.53 (279.12, 579.03)	Cuba	1.99 (1.93, 2.04)	Andorra	239.14 (151.52, 352.80)	Saint Kitts and Nevis	1.42 (1.23, 1.61)	China	257.45 (142.72, 429.32)	Sweden	2.79 (2.64, 2.93)
The bottom ten countries or territories ranked by ASR or AAPC	Uzbekistan	21.20 (11.45, 33.54)	Belarus	-4.73 (-4.99, -4.46)	Kuwait	14.45 (9.32, 21.72)	Belarus	-4.10 (-4.21, -3.99)	Kuwait	8.89 (5.68, 13.58)	Belarus	-5.15 (-5.41, -4.90)
	Bulgaria	21.67 (13.14, 33.68)	Uzbekistan	-3.64 (-3.82, -3.46)	Uzbekistan	16.68 (7.49, 27.87)	Uzbekistan	-3.93 (-4.07, -3.79)	Bulgaria	9.14 (5.54, 14.31)	Republic of Moldova	-4.48 (-4.62, -4.33)
	Kuwait	22.20 (14.54, 33.01)	Kyrgyzstan	-3.59 (-3.85, -3.34)	Turkmenistan	20.46 (10.57, 32.29)	Kyrgyzstan	-3.67 (-3.95, -3.39)	Estonia	9.23 (4.40, 17.73)	Uzbekistan	-4.00 (-4.15, -3.86)
	Estonia	23.50 (11.97, 40.53)	Turkmenistan	-3.46 (-3.58, -3.34)	Bulgaria	22.21 (12.93, 34.77)	Turkmenistan	-3.51 (-3.56, -3.45)	Republic of Moldova	10.63 (6.33, 17.34)	Kyrgyzstan	-3.95 (-4.02, -3.87)
	Turkmenistan	25.61 (14.83, 39.74)	Republic of Moldova	-3.38 (-3.67, -3.10)	Georgia	25.16 (5.66, 48.15)	Republic of Moldova	-3.35 (-3.73, -2.96)	Saint Vincent and the Grenadines	10.70 (5.87, 18.21)	Turkmenistan	-3.72 (-3.94, -3.50)

© 2025. Kang J et al. Published online at http://www.aginganddisease.org/EN/10.14336/AD.2025.1044

SUPPLEMENTARY DATA

	Georgia	29.13 (9.44, 55.59)	Bulgaria	-2.77 (-2.88, -2.65)	Estonia	26.93 (13.26, 45.52)	Armenia	-2.79 (-3.26, -2.31)	Guyana	10.73 (6.82, 16.23)	Ukraine	-3.62 (-4.18, -3.06)
	Montenegro	30.77 (16.11, 55.83)	Ukraine	-2.74 (-3.34, -2.14)	Montenegro	29.70 (15.00, 53.28)	Bulgaria	-2.79 (-2.92, -2.65)	Uzbekistan	11.37 (6.83, 17.42)	Romania	-2.56 (-2.69, -2.43)
	Belarus	31.62 (17.57, 53.57)	Romania	-2.67 (-2.89, -2.46)	Iraq	31.27 (20.34, 45.65)	Ukraine	-2.48 (-3.10, -1.85)	Antigua and Barbuda	11.78 (7.15, 18.82)	Bulgaria	-2.55 (-2.68, -2.42)
	Latvia	32.23 (17.75, 52.70)	Lithuania	-2.29 (-2.36, -2.23)	Brunei Darussalam	32.30 (8.50, 67.88)	Equatorial Guinea	-2.44 (-2.55, -2.32)	Belarus	11.83 (6.31, 20.62)	Lithuania	-2.52 (-2.59, -2.45)
	Republic of Moldova	33.71 (19.98, 53.28)	Equatorial Guinea	-2.28 (-2.34, -2.22)	Azerbaijan	35.08 (17.94, 55.74)	Romania	-2.43 (-2.61, -2.26)	Bahamas	12.39 (7.95, 18.73)	Equatorial Guinea	-2.27 (-2.33, -2.21)
**YLDs**												
The top ten countries or territories ranked by ASR or AAPC	Papua New Guinea	55.57 (32.11, 84.65)	Norway	3.92 (3.70, 4.14)	China	37.16 (19.27, 60.40)	Norway	3.53 (3.41, 3.65)	Somalia	30.63 (11.90, 60.63)	Germany	4.44 (4.17, 4.72)
	China	51.20 (26.54, 84.65)	Denmark	3.62 (3.51, 3.72)	Democratic People's Republic of Korea	35.81 (18.54, 59.06)	Israel	3.01 (2.81, 3.21)	South Africa	30.17 (13.32, 56.65)	Czechia	4.43 (3.81, 5.05)
	Nepal	47.78 (27.01, 76.14)	Czechia	2.88 (2.33, 3.44)	Papua New Guinea	35.34 (18.55, 56.43)	Denmark	2.65 (2.52, 2.78)	Lesotho	27.54 (10.37, 56.19)	Norway	3.89 (3.48, 4.30)
	Democratic People's Republic of Korea	44.09 (24.01, 70.53)	Israel	2.58 (2.45, 2.71)	Nepal	30.69 (15.56, 51.34)	Czechia	2.24 (1.72, 2.78)	Canada	27.40 (13.87, 45.35)	Netherlands	3.69 (3.42, 3.97)
	India	42.35 (21.93, 74.15)	Netherlands	2.53 (2.29, 2.77)	India	26.74 (13.43, 46.88)	Cuba	1.85 (1.72, 1.98)	Papua New Guinea	27.26 (13.76, 46.97)	Finland	3.31 (3.05, 3.57)
	Somalia	42.14 (17.11, 80.94)	Germany	2.41 (2.16, 2.66)	Myanmar	25.22 (12.90, 41.70)	Finland	1.66 (1.28, 2.03)	Nepal	25.22 (12.87, 41.96)	Denmark	3.24 (3.01, 3.46)
	South Africa	37.86 (16.91, 71.64)	Sweden	2.36 (2.28, 2.44)	Thailand	24.54 (12.21, 41.55)	Luxembourg	1.54 (1.24, 1.84)	Australia	24.36 (13.53, 37.98)	Switzerland	3.20 (3.05, 3.36)
	Lesotho	37.85 (15.42, 75.38)	Finland	2.19 (1.80, 2.58)	Australia	23.85 (12.46, 37.16)	Netherlands	1.51 (1.27, 1.75)	Rwanda	23.29 (8.46, 47.38)	Israel	3.11 (2.93, 3.29)
	Canada	37.13 (20.05, 58.92)	Canada	2.06 (1.85, 2.26)	Andorra	21.80 (10.81, 37.47)	Germany	1.44 (1.19, 1.70)	Burundi	23.21 (8.61, 46.91)	Austria	2.92 (2.63, 3.22)
	Bhutan	36.10 (20.31, 57.09)	Switzerland	2.05 (1.74, 2.36)	France	21.63 (11.04, 34.15)	Saint Kitts and Nevis	1.43 (1.17, 1.69)	Greenland	23.20 (9.31, 42.03)	Sweden	2.80 (2.59, 3.01)
The bottom ten countries or territories ranked by ASR or AAPC	Uzbekistan	1.92 (0.77, 3.54)	Belarus	-4.64 (-4.92, -4.37)	Kuwait	1.52 (0.50, 2.98)	Belarus	-4.11 (-4.29, -3.93)	Kuwait	0.82 (0.43, 1.36)	Belarus	-5.01 (-5.24, -4.79)
	Bulgaria	1.98 (0.87, 3.56)	Kyrgyzstan	-3.59 (-3.86, -3.32)	Uzbekistan	1.86 (0.57, 3.72)	Uzbekistan	-3.90 (-4.20, -3.61)	Bulgaria	0.85 (0.44, 1.42)	Republic of Moldova	-4.52 (-4.83, -4.21)
	Kuwait	1.99 (0.93, 3.52)	Uzbekistan	-3.58 (-3.75, -3.41)	Turkmenistan	2.03 (0.68, 4.01)	Kyrgyzstan	-3.66 (-3.94, -3.38)	Estonia	0.86 (0.36, 1.71)	Kyrgyzstan	-3.90 (-4.06, -3.75)

© 2025. Kang J et al. Published online at http://www.aginganddisease.org/EN/10.14336/AD.2025.1044

SUPPLEMENTARY DATA

Estonia	2.15 (0.82, 4.27)	Turkmenistan	-3.39 (-3.49, -3.28)	Bulgaria	2.26 (0.26, 5.52)	Turkmenistan	-3.44 (-3.57, -3.32)	Republic of Moldova	0.98 (0.50, 1.71)	Uzbekistan	-3.90 (-4.18, -3.62)
Turkmenistan	2.31 (0.96, 4.18)	Republic of Moldova	-3.37 (-3.62, -3.12)	Georgia	2.47 (0.79, 5.10)	Republic of Moldova	-3.35 (-3.70, -3.01)	Saint Vincent and the Grenadines	0.99 (0.47, 1.84)	Ukraine	-3.78 (-4.47, -3.08)
Georgia	2.63 (0.62, 5.60)	Bulgaria	-2.73 (-2.88, -2.58)	Estonia	2.76 (0.68, 6.15)	Bulgaria	-2.75 (-2.93, -2.57)	Guyana	0.99 (0.53, 1.61)	Turkmenistan	-3.60 (-3.88, -3.32)
Montenegro	2.81 (0.97, 5.80)	Ukraine	-2.67 (-3.22, -2.12)	Montenegro	2.81 (0.92, 5.64)	Armenia	-2.44 (-3.52, -1.36)	Uzbekistan	1.05 (0.52, 1.76)	Bulgaria	-2.48 (-2.64, -2.33)
Belarus	2.85 (1.12, 5.43)	Romania	-2.64 (-2.85, -2.44)	Iraq	2.92 (0.48, 7.29)	Romania	-2.43 (-2.63, -2.23)	Antigua and Barbuda	1.09 (0.57, 1.84)	Romania	-2.47 (-2.86, -2.08)
Latvia	2.90 (1.12, 5.69)	Lithuania	-2.28 (-2.38, -2.17)	Brunei Darussalam	3.14 (0.89, 6.31)	Equatorial Guinea	-2.42 (-2.61, -2.24)	Belarus	1.10 (0.51, 2.05)	Lithuania	-2.45 (-2.64, -2.27)
Republic of Moldova	3.04 (1.30, 5.74)	Equatorial Guinea	-2.25 (-2.30, -2.19)	Azerbaijan	47.63 (30.50, 72.50)	Ukraine	-2.41 (-3.02, -1.80)	Bahamas	1.14 (0.61, 1.86)	Thailand	-2.26 (-2.36, -2.17)

Abbreviation: ASR, age-standardized rate; YLDs, years lived with disability; COPD, chronic obstructive pulmonary disease; HF, heart failure; AAPC, average annual percentage change; UI, uncertainty interval; CI, confidence interval.

© 2025. Kang J et al. Published online at http://www.aginganddisease.org/EN/10.14336/AD.2025.1044
